# Plakilactones G and H from a marine sponge. Stereochemical determination of highly flexible systems by quantitative NMR-derived interproton distances combined with quantum mechanical calculations of ^13^C chemical shifts

**DOI:** 10.3762/bjoc.9.331

**Published:** 2013-12-30

**Authors:** Simone Di Micco, Angela Zampella, Maria Valeria D’Auria, Carmen Festa, Simona De Marino, Raffaele Riccio, Craig P Butts, Giuseppe Bifulco

**Affiliations:** 1 Dipartimento di Farmacia, Università di Salerno, via Giovanni Paolo II 132, 84084 Fisciano (SA), Italy; 2 Dipartimento di Farmacia, Università di Napoli “Federico II”, via D. Montesano 49, 80131 Napoli, Italy; 3 Department of Chemistry, University of Bristol, Cantocks Close, BS8 1TS Bristol, United Kingdom

**Keywords:** chemical shift calculations, DFT, NMR spectroscopy, quantitative NOE, stereochemical determination of flexible systems

## Abstract

In this paper the stereostructural investigation of two new oxygenated polyketides, plakilactones G and H, isolated from the marine sponge *Plakinastrella mamillaris* collected at Fiji Islands, is reported. The stereostructural studies began on plakilactone H by applying an integrated approach of the NOE-based protocol and quantum mechanical calculations of ^13^C chemical shifts. In particular, plakilactone H was used as a template to extend the application of NMR-derived interproton distances to a highly flexible molecular system with simultaneous assignment of four non-contiguous stereocenters. Chemical derivatization and quantum mechanical calculations of ^13^C on plakilactone G along with a plausible biogenetic interconversion between plakilactone G and plakilactone H allowed us to determine the absolute configuration in this two new oxygenated polyketides.

## Introduction

In recent years the quantum mechanical (QM) calculation of NMR parameters [1–6] has been demonstrated to be a valid tool for the stereostructural determination of organic compounds [7–17], especially for high flexible systems. Recently, an additional method has been proposed for the relative configuration assignment based on experimental interproton distances derived from a quantitative and accurate NOEs analysis [18]. These quantitatively measured NOEs have been initially developed for the stereochemical assignments of rigid molecular frameworks, as the NOE analysis is complicated due to equilibriums between multiple conformers, which are present in highly flexible molecules. Recently, the quantitative NOE-based method has been extended to relatively flexible compounds, and the reliability of the approach for the analysis of multiconformational systems was shown [19–20].

**Figure 1:**
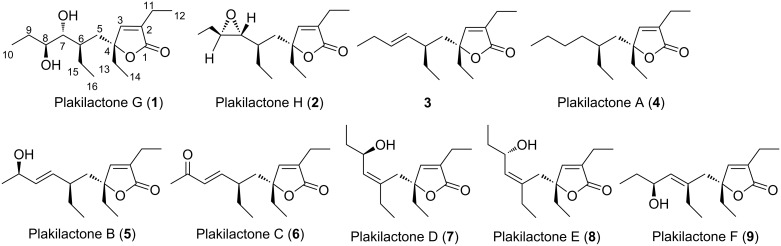
Plakilactones G and H, new oxygenated polyketides from *Plakinastrella mamillaris*, and their congeners previously reported.

Due to the huge chemical variety of secondary metabolites from natural sources, the identification of the configuration of highly flexible compounds is still a great challenge. We recently demonstrated that an integrated approach combining quantitative NOE-based protocol in parallel with the quantum mechanical calculation of ^13^C chemical shifts leads to a better discrimination of stereochemical configurations of a rigid natural product scaffold [21]. In the present contribution, we propose to extend our integrated approach to the substantially more challenging stereochemical configurations of two new conformationally flexible oxygenated polyketides, plakilactone G (**1**) and H (**2**) (Figure 1), isolated from a Fiji collection of the marine sponge *Plakinastrella mamillaris*.

## Results and Discussion

### Isolation and determination of the constitution of plakilactones G and H

**Figure 2:**
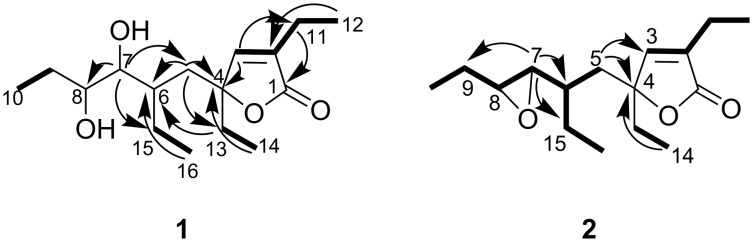
COSY connectivities (bold bonds) and selected HMBC (arrows) correlations for plakilactones G (**1**) and H (**2**).

The chloroform extract from the Kupchan partitioning procedure [22] on the lyophilized material (171 g) of *P. mamillaris* Kirkpatrick, 1900 (Homoscleromorpha) afforded plakilactones B–F, previously reported as a new chemotype of PPARγ modulators [23], together with two new oxygenated polyketides, plakilactones G (**1**) and H (**2**). As depicted in Figure 1, **1** and **2** share with other members of this family a large portion of their chemical scaffold including the γ-lactone moiety and the ethyl-branched side chain. Even if the absolute configuration at C-4 and C-6 has been previously determined for plakilactone A (**4**) and notably for the corresponding 7,8-dehydroderivative (**3**) [23], that is likely the biosynthetic precursor of all side-chain-oxidized derivatives belonging to this family, this information was not considered for the validation of our protocol and all four stereocenters for plakilactones G and H have been investigated.

Plakilactone G (**1**) was isolated as a colorless oil, [α]_D_^25^ −75.5 (*c* 0.11, CHCl_3_), and had a formula of C_16_H_28_O_4_ inferred from high resolution mass spectrum (HRMS–ESI). ^1^H and ^13^C NMR data (Table 1) indicated the presence of four ethyl groups, one methine, one methylene, one substituted double bond, one quaternary and two secondary oxygenated carbons and one acyl group. The acyl carbon signal at 175.9 ppm (C-1), along with the oxygenated carbon resonance at δ_C_ 91.9 (C-4) suggested the presence of a lactone. The olefinic methine carbon at δ_C_ 153.5 (C-3) with the quaternary carbon at δ_C_ 136.6 (C-2) completed the five-membered α,β-unsaturated lactone ring. The linkage of an ethyl side chain at C-2, suggested by the long range allylic coupling between protons H-3 and H_2_-11, was supported by the diagnostic HMBC correlations H-3/C-11 and H_3_-12/C-2 (Figure 2). A second isolated ethyl system was linked at C-4 on the basis of the HMBC correlation H_3_-14/C-4. Due to the fortuitous coincidence of the chemical shift of some protons in the side chain (e.g. H-7 and H-8; H-6 and H_2_-15) and the absence of a detectable homonuclear coupling between H-6 and H-7 protons, the analysis of the COSY spectrum only allowed for the identification of some separated subunits, which were eventually connected on the basis of diagnostic long-range correlations from the HMBC spectrum. In detail, the long range correlation H-7/C-8 implied the C-7/C-8 linkage; the correlation H_3_-16/C-6 supported the attachment of an ethyl group at C-6; the correlations H-7/C-15 and C-5 connected C-6 to C-7 (Figure 2). Finally the dihydroxylated C_8_ side chain was linked to C-4 on the basis of HMBC correlations H-5/C4 and C-13, leading to the constitution as depicted in Figure 2.

**Table 1:** ^1^H and ^13^C NMR data (500 and 125 MHz, CD_3_OD) of plakilactones G (**1**) and H (**2**).

	**1**			**2**		

position	δ_H_^a^	δ_C_	HMBC	δ_H_^a^	δ_C_	HMBC

1	–	175.9		–	175.5	
2	–	136.6		–	136.6	
3	7.11 br t (1.5)	153.5	C1, C2, C4, C11	7.12 br t (1.2)	153.0	C1, C2, C4, C11
4	–	91.9		–	90.7	
5	2.04 d (14.7) 1.64 dd (6.0, 14.7)	36.6	C3, C4, C6, C7, C13, C15 C3, C4, C6, C13, C15	1.95 dd (5.2, 14.7) 1.87 ovl	40.0	C3, C4, C6, C7, C13, C15 C3, C4, C6, C7, C13, C15
6	1.43 ovl	37.4		0.91 ovl	39.5	C4, C7
7	3.33^b^	75.3	C5, C8, C15	2.50 dd (2.0, 8.0)	63.3	C6, C9, C15
8	3.32^b^	73.9	C7	2.69 ddd (2.0, 5.6, 7.5)	61.6	C9
9	1.76 m 1.34 m	27.4	C10 C8, C10	1.54 m	26.0	C8, C10
10	1.00 t (7.4)	10.1	C8, C9	0.98 t (7.5)	9.9	C8, C9
11	2.28 q (7.5)	19.2	C1, C2, C3, C12	2.26 q (7.5)	19.3	C1, C2, C3, C12
12	1.17 t (7.5)	12.1	C2, C11	1.16 t (7.5)	12.3	C2, C11
13	1.85 m	32.1	C3, C4, C5, C14	1.86 ovl, 1.84 m	31.8	C5, C14
14	0.81 t (7.5)	7.8	C4, C13	0.81 t (7.3)	7.8	C4, C13
15	1.43 ovl	26.2	C6	1.44 m 1.39 m	26.9	C5, C6, C7, C16 C5, C6, C7, C16
16	0.85 t (6.8)	11.6	C6, C15	0.91 t (7.5)	11.8	C6, C15

^a^Coupling constants are in parentheses and given in Hertz. ^1^H and ^13^C assignments aided by COSY, HSQC and HMBC experiments. ^b^Overlapped with solvent signal; ovl: overlapped with other signals.

Plakilactone H (**2**) was isolated as a colorless oil, [α]_D_^25^–47.9 (*c* 0.07, CHCl_3_) and showed a molecular formula of C_16_H_26_O_3_ as deduced by HRMS–ESI analysis. The proton and carbon signals of the 2,4-diethyl γ-lactone were almost identical to those of the corresponding part of plakilactone G (**1**), whereas differences were observed in the C-8 side chain. Two mutually coupled signals at δ_H_ 2.50 (dd, *J* = 2.0 and 8.0 Hz) and 2.69 (ddd, *J* = 2.0, 5.6, 7.5 Hz), observed in the ^1^H NMR spectrum, were found to correlate in the HSQC spectrum with two oxygenated carbons at δ_C_ 63.3 and 61.6, respectively, and were assigned to an epoxy ring. The localization of the epoxy functionality at C-7 and the structure (Figure 2) of the side chain was easily inferred from the analysis of the COSY spectrum and confirmed by key HMBC correlations (Table 1 and Figure 2). The connection of the side chain to C-4 was established by long-range couplings observed between the two diasterotopic methylene protons at C-5 and the C-3 and C-4 carbons of the lactone ring. Therefore the constitution of plakilactone H (**2**) was determined as shown in Figure 2.

### Determination of the relative configuration of plakilactone H (**2**)

The better dispersion of proton resonances in the ^1^H NMR spectrum of plakilactone H inclined us to first address the configurational assignment of plakilactone H.

**Figure 3:**
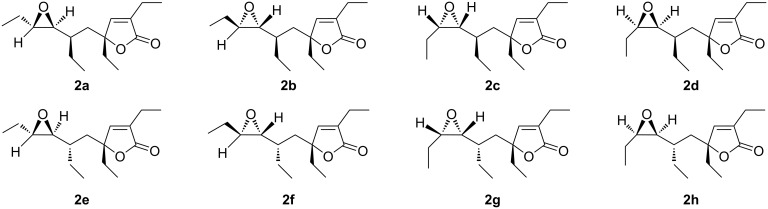
Molecular structure of the eight possible diastereoisomers of plakilactone H (**2**).

Molecular dynamics and Monte Carlo conformational search calculations were performed on all possible diastereoisomers of **2** (Figure 3) by using the MMFFs [24] force field (MacroModel software package [25]) in the presence of chloroform (continuum model). Over 200 conformers were found for each of the stereoisomers for **2a–h** (see Experimental), and their geometries were optimized at the DFT theoretical level by using the MPW1PW91 functional and 6-31G(d) [26] basis set (Gaussian 09 Software Package [27]). From the DFT-optimized geometries interproton distances were calculated, accounting the Boltzmann-weighted average derived from the energies of the single conformers.

For the analysis, we applied the previously described method [18–21], firstly recording different 1D NOESY spectra, irradiating at diverse resonances. In particular, the NOE coupling between vicinal protons H-15b–H-16 were chosen as the reference NOE for the 1D NOESY data set and the derived distance was adjusted for each stereoisomer in order to get the lowest MAE. To narrow the number of diastereoisomers, we initially analysed the relative configuration of C-7 and C-8, to disclose if the substituents of the epoxide are *cis* or *trans*-configured.

The observed absolute differences for calculated versus NOE-derived distances/calculated distances (Table 2) suggested a *trans*-configuration for the epoxide (MAE of 4.7% vs 20.7% for *cis*-isomer) ring. The next step was the analysis of the four diastereoisomers **2a**,**b**,**e**,**f** endowed with the epoxide moiety in a *trans*-configuration, by comparing the experimental vs the calculated distances (Table 3). In Table 3 only a subset of all values was used for the stereochemical structure elucidation, more specifically, the values where DFT-calculated interproton distances for **2a**,**b**,**e**,**f** differed by more than 0.03 Å (≈ 1%) from each other. The data reported in Table 3 clearly show that the diastereoisomer **2b** represents the best fit with the experimentally derived distances (MAE 4.1% and standard deviation (STD) 5.0%). The stereoisomers **2a**, **2e** and **2f** poorly agree with the NOE-derived distances. The maximum error shown by the calculated distances for **2b** is 9.4%, whereas the other stereoisomers have at least three calculated distances with an associated error higher than 10%. The largest errors (>10%) are relative to protons around the stereocenters under investigation. For **2a**, we observed an error of 25.3% for the distance between H-16–H-8 and 12.2% for the protons H-3–H-13a. The calculated interproton distance H-3–H-6 presents an error of 14.7%, whereas the distance H-15b–H-6 has an error of 11.8%. It is noteworthy that **2a** has the key distance between H-6 and H-8 with a quite high error of 9.8%. Concerning the diastereoisomer **2e**, largest errors are observed for the distances of H-3 with H-6 (11.3%) and H-14 (17.2%). The H-6–H-15b distance has an error of 12.5%, and H-6 shows a large deviation with H-5b of 11.5%. As for **2a**, a huge error (39.7%) for the H-16–H-8 distance was found in diasteroisomer **2f**. In addition, H-5b presents large deviations from the experimental about distances with H-6 (19.0%) and H-7 (10.6%) and a deviation >10% for the interproton distances of H-15a–H-8 (13.0%), H-7–H-10 (11.4%) and H-15a–H-16 (10.5%) was also observed.

**Table 2:** Comparison of interproton distances determined by NOEs for plakilactone H (**2**) in CDCl_3_ with DFT-calculated values for **2b** and **2c**. Values in bold were used to calibrate the NOEs.

			**2b**		**2c**	

proton		exp R_NOE_ (Å)	R_calcd_ (Å)	ABS % error^a^	R_calcd_ (Å)	ABS % error^a^

H16	H15b	**2.75**	**2.75**	–	**2.75**	–
H8	H9	2.68	2.73	1.8%	2.72	1.5%
H8	H10	3.27	3.02	7.9%	2.95	10.1%
H8	H6	2.32	2.44	4.9%	3.80	48.4%
H7	H9	2.65	2.71	2.1%	3.93	38.7%
H7	H10	3.60	3.39	6.1%	4.71	26.8%
H7	H6	2.94	3.03	2.9%	3.07	4.2%
MAE^b^				*4.7%*		*20.7%*
STD				*2.9%*		*17.3%*

^a^|% error| = |R_calcd_ − R_NOE_|/[(R_calcd_ + R_NOE_)/2], absolute differences for calculated versus NOE-derived distances/calculated distances. ^b^MAE = Σ[% error]/*n*.

**Table 3:** Comparison of interproton distances determined by NOEs for plakilactone H (**2**) in CDCl_3_ with DFT-calculated values for **2a**,**b**,**e**,**f**. Values in bold were used to calibrate the NOEs.

			**2a**		**2b**		**2e**		**2f**	

proton		exp R_NOE_ (Å)	R_calcd_ (Å)	ABS % error^a^	R_calcd_ (Å)	ABS % error^a^	R_calcd_ (Å)	ABS % error^a^	R_calcd_ (Å)	ABS % error^a^

H16	H15b	**2.75**	**2.75**	0.0%	**2.75**	0.0%	**2.71**	1.5%	**2.72**	1.1%
H16	H8	3.32	4.28	25.3%	3.37	1.5%	3.40	2.4%	4.97	39.7%
H3	H11	3.14	3.33	5.9%	3.37	7.1%	3.27	4.1%	3.26	3.9%
H3	H5b	2.90	2.81	3.2%	2.74	5.7%	2.84	2.2%	2.76	5.0%
H3	H13a	2.85	3.22	12.2%	2.86	0.4%	3.00	5.0%	2.89	1.3%
H3	H12	3.20	3.03	5.5%	3	6.5%	3.01	6.3%	3.04	5.2%
H3	H14	3.11	3.08	1.0%	3.3	5.9%	3.70	17.2%	3.43	9.8%
H3	H6	3.22	3.73	14.7%	3.13	2.8%	3.61	11.3%	3.08	4.6%
H8	H9	2.68	2.71	1.1%	2.68	0.0%	2.66	0.7%	2.66	0.7%
H8	H10	3.21	3.03	5.8%	3.09	3.8%	3.00	6.9%	3.01	6.5%
H8	H6	2.32	2.56	9.8%	2.44	5.0%	2.50	7.6%	2.44	5.2%
H7	H5b	2.64	2.77	4.8%	2.79	5.5%	2.68	1.5%	2.37	10.6%
H7	H9	2.65	2.71	2.2%	2.77	4.4%	2.72	2.6%	2.66	0.4%
H7	H15a	2.73	2.97	8.4%	2.59	5.3%	2.82	3.2%	2.90	6.0%
H7	H10	3.54	3.76	6.0%	3.39	4.3%	3.44	2.9%	3.97	11.4%
H7	H16	3.44	3.21	6.9%	3.39	1.5%	3.48	1.1%	3.44	0.1%
H7	H6	2.94	2.69	8.9%	3.03	3.0%	2.91	1.1%	2.94	0.1%
H5b	H13a	2.67	2.82	5.5%	2.52	5.8%	2.75	2.9%	2.54	4.9%
H5b	H6	3.23	2.94	9.4%	2.98	8.1%	2.88	11.5%	2.67	19.0%
H5b	H14	3.04	2.99	1.7%	3.28	7.6%	3.04	0.2%	3.35	9.8%
H15b	H6	2.97	2.64	11.8%	2.91	2.0%	2.62	12.5%	2.69	9.9%
H15a	H8	3.44	3.24	6.0%	3.66	6.2%	3.38	1.8%	3.92	13.0%
H9	H10	2.74	2.75	0.3%	2.75	0.3%	2.71	1.2%	2.72	0.8%
H15a	H16	3.02	2.75	9.4%	2.75	9.4%	2.71	10.9%	2.72	10.5%
H12	H11	2.76	2.75	0.3%	2.75	0.3%	2.71	1.8%	2.72	1.4%
MAE^b^				*6.6%*		*4.1%*		*4.8%*		*7.2%*
STD				*8.7%*		*5.0%*		*6.7%*		*11.1%*

^a^|% error| = |R_calcd_ − R_NOE_|/[(R_calcd_ + R_NOE_)/2], absolute differences for calculated versus NOE-derived distances/calculated distances. ^b^MAE = Σ[% error]/*n*.

In previous papers [18–21], describing the accurate measurement of interproton distances from NOE, it was established that the expected MAE and STD are both around 5% or less for rigid and simple flexible molecules, and substantial individual errors of more than 10% are indicative of incorrect assignments. In this more complex, flexible molecule we found a MAE of 4.1% and a STD of 5.0% for **2b**, which are in line with correct assignments obtained in our earlier studies. For example, the obtained MAE for **2b** is identical to the MAE obtained for the previously reported test case of strychnine in CDCl_3_ [18]. On the other hand, the obtained MAE and STD for **2a**,**e**,**f** are all out of the expected range, although **2e** has a nearly acceptable MAE value, the range of error (as represented by an STD of 6.7%) is too wide, and five of the individual distances have errors of ≥10% (underlined values in Table 3).

The results derived from NOE analysis were confirmed by QM calculation of ^13^C chemical shifts. On the refined geometries at the DFT theoretical level for **2a**,**b**,**e**, and **f**, ^13^C chemical shift values were calculated by using the MPW1PW91 functional and the 6-31G(d,p) basis set [26] (Gaussian 09 Software Package [27]) and taking into account the Boltzmann-weighted average derived from the energies of the single conformers. The analysis was carried out with linear regression analysis by using values as intercept and slope, which were obtained at the same level of theory for a set of known natural compounds [26]. Moreover, we considered the diagnostic carbons and calculated the difference with the experimental values that were re-acquired and reassigned in CDCl_3_ (Supporting Information File 1, Table S1), to avoid the introduction of explicit solvent molecules in the calculations, as required for a polar and protic solvent such as methanol. Comparison of predicted ^13^C chemical shifts for **2a**,**b**,**e**,**f** with the experimental values (Table 4) suggests the best fit with the experimental data for stereoisomer **2b**. In particular, **2a** presents a MAE more than twice the value found for **2b**. The MAE of **2e** is almost twice the value of **2b**. By analysing the |Δδ| for carbon atoms around C-6, we find largest errors for the calculated ^13^C chemical shifts of **2e**, except for C-14 (Table 4). In detail, for C-5 and C-13 of **2e** the |Δδ| are 1.5 and 2.1, vs 1.1 and 1.0 of **2b**. For C-15 and C-16, the |Δδ| are 1.3 and 1.3 for **2e**, whereas for **2b** they are 1.3 and 0.0, respectively. Moreover, we observe large deviations from the experimental values for C-8 and C-9 of **2e**. Indeed, the |Δδ| of C-8 and C-9 are 1.7 and 1.1, compared to 0.1 and 0.7 for **2b**. A smaller difference for the MAE values calculated for **2b** and **2f** is observed. The stereoisomer **2b** shows all calculated ^13^C values falling in the proposed error limit of 2 ppm. For **2b**, we observed a maximum error of 1.5 ppm. On the other hand, we found a |Δδ| of 2.5 ppm for **2f**. Parallel with the MAE and |Δδ| analysis, we compared the calculated ^13^C chemical shifts with the experimental values by using the DP4 probability [28]. This analysis also shows that the best fit with the experimental chemical shifts are found for **2b**, which has 78.9% of DP4 probability (Table 4). The **2a**, **2e** and **2f** present low DP4 probability values: 1.5%, 3.9% and 15.7%, respectively (Table 4). The stereostructural analysis by the DFT-NMR approach agrees with the outcomes obtained by the accurate NOE-distance method, confirming the relative configuration of plakilactone H as in diastereoisomer **2b**.

**Table 4:** Comparison of calculated (in vacuo) vs experimental (in CDCl_3_) ^13^C NMR chemical shifts of stereoisomers **2a**,**b**,**e** and **f**.

		**2a**		**2b**		**2e**		**2f**	

carbon	δ_exp_	δ_calcd_	|Δδ|^a^, ppm	δ_calcd_	|Δδ|^a^, ppm	δ_calcd_	|Δδ|^a^, ppm	δ_calcd_	|Δδ|^a^, ppm

10	9.7	10.8	1.1	9.6	0.1	9.6	0.1	10.8	1.1
9	24.9	25.7	0.7	25.6	0.7	26.0	1.1	25.1	0.2
8	60.9	58.5	2.3	61.0	0.1	59.1	1.7	62.1	1.3
7	62.1	59.5	2.6	60.6	1.5	60.5	1.6	61.1	1.0
6	38.3	36.1	2.2	37.9	0.3	38.8	0.5	36.4	1.8
5	39.2	36.7	2.5	40.3	1.1	40.6	1.5	39.3	0.1
13	31.2	30.7	0.5	32.2	1.0	29.1	2.1	32.6	1.5
14	7.7	7.9	0.1	6.9	0.9	7.4	0.3	6.8	0.9
15	25.8	28.2	2.3	27.1	1.3	27.1	1.3	26.1	0.3
16	11.4	10.0	1.4	11.4	0.0	10.1	1.3	8.9	2.5
MAE^b^			*1.6*		*0.7*		*1.2*		*1.1*
DP4^c^			*1.5*		*78.9*		*3.9*		*15.7*

^a^|Δδ| = |δ_exp_ − δ_calcd_|, absolute differences for experimental versus calculated ^13^C NMR chemical shifts. ^b^MAE = Σ[|δ_exp_ − δ_calcd_|]/*n*. ^c^DP4 probabilities were obtained considering all the calculated chemical shifts , as proposed by Smith and Goodman [28].

### Determination of the absolute configuration

The 1,2-diol substructure in plakilactone G (**1**) allowed the configurational assignment of the C-7 and C-8 contiguous stereocenters through chemical derivatization. Thus, plakilactone G (**1**) was converted to the corresponding 7,8-*O*-isopropylidene derivative by treatment with 2,2-dimethoxypropane and a catalytic amount of *p*-TsOH. As reported in the literature [29], the difference in the chemical shifts of the methyl groups in the five membered acetonide is larger for the *cis*-isomer (Δδ 0.12–0.14) when compared to the *trans*-isomer (Δδ 0.01–0.04). The observed Δδ value of 0.10 ppm between the two methyl groups in the plakilactone G acetonide (see Experimental) points towards the *cis*-isomer allowing us to suggest a 7,8-*erythro* relative stereochemistry.

**Figure 4:**
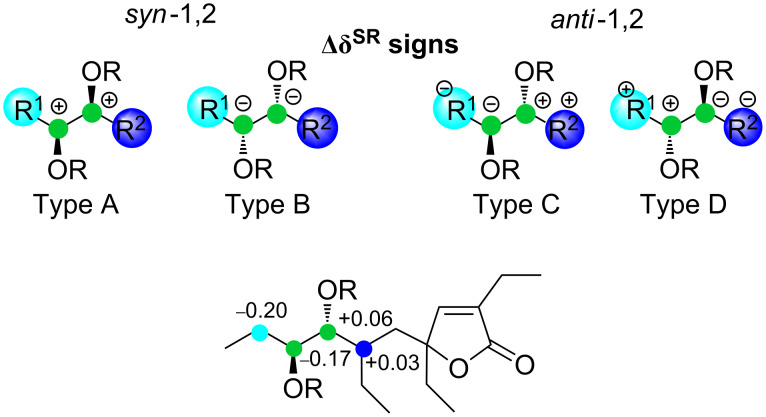
Δδ^SR^ sign distribution model for the bisMTPA esters of a 1,2-diol and absolute configuration for C-7–C-8 diol in plakilactone G (**1**).

The application of the double derivatization method with a chiral auxiliary reagent developed by Riguera [30] allows for the confirmation of the relative configuration at C-7 and C-8 and the assignment of the absolute configuration at C-7 and C-8. Through theoretical calculation and experimental data, Riguera demonstrated that bisphenylacetic acid ester derivatives of a diol with two asymmetric carbons have a specific and distinctive distribution of Δδ^SR^ signs, determined by a combined anisotropy effect of the two auxiliares. The Δδ^SR^ distribution model for a bisMTPA derivative of an acyclic 1,2-diol is shown in Figure 4. Thus, esterification of plakilactone G (**1**) with (−)- and (+)-MTPACl in pyridine led to bisMTPA derivatives which Δδ^SR^ distribution is reported in Figure 4. The observed sign distribution model is consistent with *anti*-1,2-diol type C. Therefore the 7*R*,8*S* configuration is assigned. Notably, considering the plausible biogenetic interconversion of an epoxide and a diol, the above absolute configuration at C-7 and C-8 of plakilactone G is in full agreement with the *trans*-epoxide **2b**.

**Figure 5:**
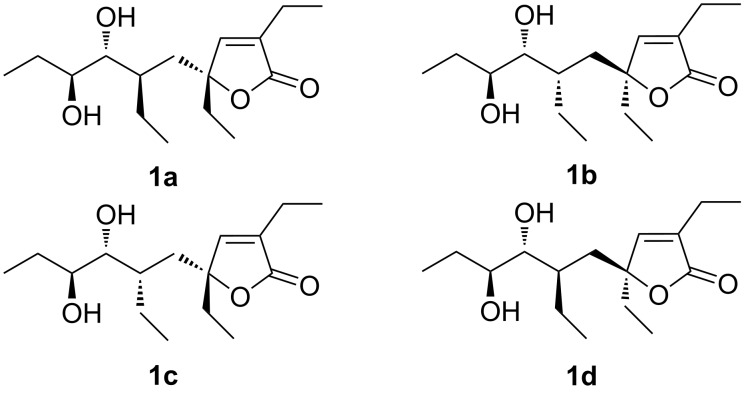
Molecular structure of the four possible diastereoisomers of plakilactone G (**1**).

Having assigned the absolute configuration at C-7 and C-8, we tried to elucidate the absolute configuration at C-4 and C-6 on plakilactone G (**1**). The first step was the conformational search of the four possible diastereoisomers (**1a**–**d** in Figure 5), obtained with a fixed 7*R*,8*S* configuration, by using molecular dynamics (400, 600 and 800 K) and MonteCarlo Multiple Minimum method (MacroModel package [25], see Experimental).

The geometries of all the significant conformers of **1a**–**d** were subsequently optimised at DFT level by using the MPW1PW91 functional and the 6-31G(d) basis set. Unfortunately, the application of quantitative NOEs method was limited by severe overlapping in many crucial proton resonances (e.g. 1.38–1.52 ppm; 3.46–3.49 ppm, Supporting Information File 1, Table S1) rendering difficult the selective irradiation and the integration of NOE peaks. Thus, many experimental NOE intensities had to be ignored, resulting in MAE and STD values higher than expected for a quantitative NOE-distance investigation of small molecules in our experience. However, the analysis of the limited number of reliable NOEs gave distances (Supporting Information File 1, Table S4) which fitted best with stereoisomer **1a** as the structure of plakilactone G (MAE of 6.8% and STD of 8.8%). For **1b**–**d** substantially larger MAE and STD values were observed, in particular for **1c** and **1d**: 8.1% and 12.5% (**1b**), 13.8% and 19.9% (**1c**); 11.3 % and 17.0% (**1d**).

Due to the lack of a definitive fit for the NOE-distance data we relied more heavily on the QM calculations of the ^13^C chemical shifts in case of the stereostructural investigation of C-4 and C-6 of plakilactone G (**1**). On the obtained geometries at DFT theoretical level, single point calculation of the ^13^C chemical shifts were performed by using the same functional and the 6-31G(d,p) basis set. The final ^13^C chemical shift values for **1a**–**d** were derived taking into account the Boltzmann weighted average based on the energies of the single conformers for each stereoisomer. The obtained ^13^C chemical shifts were compared with the experimental data by considering the diagnostic carbons and calculating the difference with the experimental values (|Δδ|) and the relative MAE (Table 5).

**Table 5:** Comparison of calculated (in vacuo) vs experimental (in CDCl_3_) ^13^C NMR chemical shifts of stereoisomers **1a**–**d**.

		**1a**		**1b**		**1c**		**1d**	

carbon	δ_exp_	δ_calcd_	|Δδ|^a^, ppm	δ_calcd_	|Δδ|^a^, ppm	δ_calcd_	|Δδ|^a^, ppm	δ_calcd_	|Δδ|^a^, ppm

9	24.0	21.6	2.4	26.0	2.0	27.5	3.5	22.1	1.9
8	73.6	72.4	1.2	74.6	0.9	73.0	0.6	70.7	2.9
7	75.6	76.1	0.5	71.3	4.3	79.1	3.5	76.4	0.8
6	35.6	36.0	0.3	37.3	1.7	34.2	1.4	34.0	1.6
5	36.2	39.2	3.1	36.9	0.8	37.4	1.2	36.8	0.6
4	89.6	87.1	2.5	87.1	2.5	88.8	0.8	87.4	2.2
13	30.9	30.3	0.5	28.0	2.9	32.3	1.4	32.7	1.8
15	25.3	24.2	1.0	22.6	2.7	20.4	4.8	24.5	0.8
16	10.7	9.6	1.1	11.1	0.4	11.7	1.0	10.2	0.5
MAE^b^			*1.4*		*2.0*		*2.0*		*1.5*
DP4^c^			*62.9*		*1.9*		*5.4*		*29.7*

^a^|Δδ| = |δ_exp_ − δ_calcd_|, absolute differences for experimental versus calculated ^13^C NMR chemical shifts. ^b^MAE = Σ[|δ_exp_ − δ_calcd_|]/*n*. ^c^DP4 probabilities were obtained considering all the calculated chemical shifts, as proposed by Smith and Goodman [28].

The comparison of ^13^C chemical shifts with the experimental data suggests that **1a** presents the best agreement with the experimental values. The stereostructural hypotheses **1b** and **1c** are unlikely given their substantially larger MAE values, whereas **1a** and **1d** both show comparable MAE values. We also applied the DP4 probability analysis proposed by Smith and Goodman [28], which strongly suggests that stereoisomer **1a** presents the best agreement with the experimental data set. Indeed, **1a** has a DP4 probability of 62.9% (Table 5), whereas for **1b**–**d** we found a probability of 1.9%, 5.4% and 29.7% (Table 5), respectively. Isomers **1a** and **1d** differ only in the configuration at C-4, and considering a putative interconversion between diol **1** and epoxide **2** and a common biogenetic pathway in combination with the chemical shift data and supported by the NOE-distance data, we suggest that the structure of plakilactone G is as depicted in **1a** and the absolute configuration of plakilactone H as depicted in **2b**.

## Conclusion

In this paper two new plakilactones are reported from the marine sponge *Plakinastrella mamillaris*. Plakilactone H was used as a template to set up the potential application of a combined approach of quantitative NMR-derived interproton distances and QM calculations of ^13^C chemical shifts in defining the stereostructure of highly flexible chemical scaffolds. The two independent methodologies agree and suggest the structure of plakilactone H as depicted in **2b**. It is noteworthy that the analysis of accurate NOE-derived distances, up to date, is limited to rigid and relatively flexible molecules and the extension of this methodology to a highly flexible natural product affirms the reliability of this approach to investigate multiconformational chemical systems. Moreover, for the first time, we simultaneously assigned the relative configuration of four stereocenters by using the NOE analysis. In particular, the C-4 stereocenter is not adjacent to the other stereogenic carbons, highlighting that the NOE-based method is useful to investigate the relative configuration of isolated carbons. The absolute configuration of plakilactone G (**1**) by using a combined approach of chemical derivatization and ^13^C QM calculation, is also reported. Firstly, the absolute configurations at C-7 and C-8 were determined by the double derivatization method with a chiral auxiliary reagent and the results were fully in agreement with the relative configuration of the epoxide moiety on the plakilactone H side chain. Subsequently, the absolute configuration at C-4 and C-6 was obtained through QM calculations of ^13^C chemical shifts, supported by the less satisfactory NOE-distance analysis in this case which failed to give a completely unambiguous solution. Considering the plausible biogenetic epoxide/diol interconversion, the absolute configuration of plakilactone H is also proposed as depicted in **2**.

## Experimental

*Plakinastrella mamillaris* Kirkpatrick, 1900 (order Homosclerophorida, family Plakinidae) was processed as previously reported [23]. A portion (5.1 g) of the overall 16.6 g of CHCl_3_ extract, rich in plakilactones [23,31–32] and gracilioethers, which was already available in our laboratory, was chromatographed by silica gel MPLC by using a solvent gradient system from CH_2_Cl_2_ to CH_2_Cl_2_/MeOH 1:1. Fractions eluted with CH_2_Cl_2_ (302 mg) were further purified by HPLC on a Nucleodur 100-5 C18 (5 μm; 10 mm i.d. × 250 mm) with 65% MeOH/H_2_O as an eluent (flow rate 3.5 mL/min) to give 6.3 mg of plakilactone H (**2**) (*t*_R_ 29.4 min). As described in [31], the purification of fractions eluted with CH_2_Cl_2_/MeOH 99:1 (2.0 g) furnished 15.4 mg of plakilactone G (**1**) (*t*_R_ 16.5 min).

### Characteristic data for each compound

Plakilactone G (**1**): colorless oil; [α]_D_^25^ −75.5 (*c* 0.11, CHCl_3_); ^1^H and ^13^C NMR data in CD_3_OD are given in Table 1; ESIMS *m*/*z*: [M + Na]^+^ 307.2; HRMS–ESI (*m*/*z*): [M + Na]^+^ calcd for C_16_H_28_NaO_4_, 307.1885; found, 307.1890.

Plakilactone H (**2**): colorless oil; [α]_D_^25^ −47.9 (*c* 0.07, CHCl_3_); ^1^H and ^13^C NMR data in CD_3_OD are given in Table 1; ESIMS *m/z*: [M + Na]^+^ 289.2; HRMS–ESI (*m*/*z*): [M + Na]^+^ calcd for C_16_H_26_NaO_3_, 289.1780; found, 289.1788.

**Acetonide derivative from plakilactone G (1)**. A mixture of **1** (1.1 mg), 2,2-dimethoxypropane (1.0 mL) and a catalytic amount of *p*-TsOH (4.0 mg) was stirred at room temperature for 4 h. Saturated aqueous NaHCO_3_ (1 mL) was then added, and the reaction mixture was extracted with CH_2_Cl_2_ (3 × 2 mL). The organic solvents were removed under a high vacuum, providing the acetonide derivative in quantitative yield. Selected ^1^H NMR (500 MHz, CD_3_OD) δ 7.11 (br t, *J* = 1.5 Hz, 1H, H-3), 3.89 (m, 2H, H-7 and H-8), 2.28 (q, *J* = 7.5 Hz, 2H, H_2_-11), 2.11 (dd, *J* = 3.9, 14.9 Hz, 1H, H-5a), 1.89 (dd, *J* = 3.9, 14.9 Hz, 1H, H-5b), 1.41 (s, 3H, Me), 1.31 (s, 3H, Me), 1.18 (t, *J* = 7.5 Hz, 3H, H_3_-12), 0.94 (t, *J* = 7.5 Hz, 3H, H_3_-16), 0.90 (t, *J* = 7.1 Hz, 3H, H_3_-10), 0.82 (t, *J* = 7.4 Hz, 3H, H_3_-14) ppm.

**General procedure for the preparation of bis-MTPA esters of plakilactone G (1)**. As described in [31], plakilactone G (0.5 mg) was dissolved in freshly distilled CH_2_Cl_2_ and treated with triethylamine (10 μL), (*R*)- or (*S*)-α-methoxy-α-(trifluoromethyl)phenylacetyl chloride (MTPACl) (5 μL) and a catalytic amount of 4-(dimethylamino)pyridine to obtain bis-(*S*)- or bis-(*R*)-MTPA esters, respectively. The mixture was left to stand at room temperature for 1 h, with the resulting mixture purified by silica gel column.

**Bis-(*S*)-MTPA ester**. Selected ^1^H NMR (500 MHz, CD_3_OD) δ 6.12 (br t, *J* = 1.7 Hz, 1H, H-3), 5.34 (m, 1H, H-7), 5.09 (m, 1H, H-8), 2.30 (m, 2H, H_2_-11), 2.22 (m, 1H, H-5a), 1.67 (m, 1H, H-5b), 1.47 (m, 2H, H_2_-9), 1.23 (m, 1H, H-6), 1.18 (t, *J* = 7.2 Hz, 3H, H_3_-12), 0.86 (t, *J* = 7.5 Hz, 3H, H_3_-10) ppm.

**Bis-(*R*)-MTPA ester**. Selected ^1^H NMR (500 MHz, CD_3_OD) δ 6.66 (br t, *J* = 1.5 Hz, 1H, H-3), 5.28 (m, 1H, H-7), 5.26 (m, 1H, H-8), 2.30 (m, 2H, H_2_-11), 1.69 (m, 2H, H_2_-9), 1.56 (dd, *J* = 3.8, 14.7 Hz, 1H, H-5a), 1.42 (dd, *J* = 3.8, 14.7 Hz, 1H, H-5b), 1.33 (m, 2H, H_2_-15), 1.20 (m, 1H, H-6), 1.18 (t, *J* = 7.3 Hz, 3H, H_3_-12), 0.98 (t, *J* = 7.4 Hz, 3H, H_3_-10), 0.91 (t, *J* = 6.5 Hz, 3H, H_3_-16), 0.63 (t, *J* = 7.5 Hz, 3H, H_3_-14) ppm.

### NMR experiments

Plakilactone G and H were dissolved in 0.5 mL of CDCl_3_ and transferred in 5 mm tubes under air without degassing. NMR experiments were performed at *T* = 298 K on a Varian 500 MHz VNMRS spectrometer equipped with an H{C,X}, and on a Varian 600 MHz VNMRS spectrometer equipped with an H{C,N} coldprobe. Chemical shifts (δ, ppm) are referenced to CDCl_3_ as an internal standard (δ_H_ 7.26, δ_C_ 77.2).

For the assignment in CDCl_3_ of **1**, we performed: 2D-COSY spectrum with 1024 t2 points, 128 t1 points, 0.15 s t2 acquisition time, and 4 scans; 2D-HSQC spectrum was obtained with 2048 t2 points, 256 t1 points, 0.15 s t2 acquisition time, and 4 scans; 2D-HMBC spectrum was obtained with 1024 t2 points, 128 t1 points, 0.15 s t2 acquisition time, and 20 scans. For the assignment in CDCl_3_ of **2**, we performed: 2D-COSY spectrum with 2048 t2 points, 128 t1 points, 0.3408 s t2 acquisition time, and 16 scans; 2D-HSQC spectrum was obtained with 2048 t2 points, 64 t1 points, 0.0745 s t2 acquisition time, and 16 scans; 2D-HMBC spectrum was obtained with 1024 t2 points, 256 t1 points, 0.1499 s t2 acquisition time, and 16 scans. To determine the interproton distances of **1** and **2**, 1D selective transient NOESY spectra were obtained by using 512 (for **1**) and 256 (for **2**) scans, acquisition time: 3.2768 s for **1**, 5.3248 s for **2**. For all NOESY spectra of **1** and **2**, 500 ms of mixing time and 1 s of relaxation delay were applied. Wurst2i selective shaped pulse was applied for the 1D-NOESY experiments. NMR data were processed by using MestreNova version7.

### Computational studies

In order to explore the conformational space of plakilactones G and H (**1** and **2**), we performed Molecular dynamics and Monte Carlo calculations. Molecular dynamics calculations of **1** and **2** were performed at different temperatures (400 and 600 and 800 K for 5 ns (time-step of 1.5 fs) by using the MMFFs [24] force field (MacroModel software package [25]). During the molecular dynamics, a standard constant temperature velocity–Verlet algorithm was used to integrate the equations of motions [33]. Independently from molecular dynamics, we also applied Monte Carlo Multiple Minimum (MCMM) method (10,000 steps) of the MacroModel module to explore the conformational space of **1** and **2** by using the MMFFs [24] force field. All molecular mechanics calculations were performed in chloroform (continuum model, MacroModel software package [25]). We found 254 major conformers for **1a**, 285 for **1b**, 574 for **1c** and 146 for **1d**. We found 209 major conformers for **2a**; 400 for **2b**; 284 for **2c**; 218 for **2d**; 225 for **2e**; 347 for **2f**; 254 for **2g**; 183 for **2h**.

All the obtained structures from both methods for **1a–d** and **2a–h** were minimized by using the Polak–Ribiere conjugate gradient algorithm (PRCG, 9 × 10^7^ steps, convergence threshold 0.001 kJ mol^−1^ Å^−1^). All the geometries of **1a–d** and **2a–h** presenting an energy difference ≤13 kJ/mol from the global minimum were retained and used for QM calculations. All the obtained geometries of **1a–d** and **2a–h** from molecular mechanics methods, were further refined in vacuo at the DFT theoretical level by using MPW1PW91 functional and the 6-31G(d) basis set [26] (Gaussian 09 software package) [27]. The DFT-optimized structures were used for the single-point ^13^C chemical shift calculations (in vacuo) with the same functional and the 6-31G(d,p) basis set. By the same theoretical level (MPW1PW91/6-31G(d,p)) we calculated frequencies for **1**. The calculated values of chemical shifts of **1** and **2** were referenced to the theoretical tetramethylsilane ^13^C chemical shift value (previously optimized at the DFT level), computed at the same level of theory.

## Supporting Information

File 1 Analytical data.
