# Supplementary material for: Plakilactones G and H from a marine sponge. Stereochemical determination of highly flexible systems by quantitative NMR-derived interproton distances combined with quantum mechanical calculations of 13C chemical shifts
Source: Beilstein J Org Chem. 2013 Dec 30;9:2940–9. doi: 10.3762/bjoc.9.331 (PMC3896268; doi:10.3762/bjoc.9.331)
Supplement: File 1 — Analytical data. [file Beilstein_J_Org_Chem-09-2940-s001.pdf]

Supporting Information
for
Plakilactones G and H from a marine sponge. Stereochemical determination of highly flexible systems by quantitative NMR-derived interproton distances combined with quantum mechanical calculations of ^{13}C chemical shifts

Simone Di Micco[1], Angela Zampella[2], Maria Valeria D'Auria[2], Carmen Festa[2], Simona De Marino[2], Raffaele Riccio[1], Craig P. Butts[3]* and Giuseppe Bifulco[1]*

[1]Dipartimento di Farmacia, Università di Salerno, via Giovanni Paolo II 132, 84084 Fisciano (SA), Italy, [2]Dipartimento di Farmacia , Università di Napoli "Federico II", via D. Montesano 49, 80131 Napoli, Italy and [3]Department of Chemistry, University of Bristol, Cantocks Close, BS8 1TS Bristol, United Kingdom

Email: Craig P. Butts - craig.butts@bristol.ac.uk; Giuseppe Bifulco - bifulco@unisa.it

Analytical data

Contents

Figure S1. ^{1}H NMR spectrum (500 MHz, CD_3OD) of plakilactone G (**1**).
Figure S2. COSY spectrum (500 MHz, CD_3OD) of plakilactone G (**1**).
Figure S3. HSQC spectrum (500 MHz, CD_3OD) of plakilactone G (**1**).
Figure S4. HMBC spectrum (500 MHz, CD_3OD) of plakilactone G (**1**).
Figure S5. ^{1}H NMR spectrum (500 MHz, CD_3OD) of plakilactone H (**2**).
Figure S6. COSY spectrum (500 MHz, CD_3OD) of plakilactone H (**2**).
Figure S7. HSQC spectrum (500 MHz, CD_3OD) of plakilactone H (**2**).
Figure S8. HMBC spectrum (500 MHz, CD_3OD) of plakilactone H (**2**).
Table S1. NMR Data of plakilactones G (**1**, 500 MHz, $CDCl_3$) and H (**2**, 600 MHz, $CDCl_3$)
Figure S9. ^{1}H NMR spectrum (500 MHz, $CDCl_3$) of plakilactone G (**1**).
Figure S10. COSY spectrum (500 MHz, $CDCl_3$) of plakilactone G (**1**).
Figure S11. HSQC spectrum (500 MHz, $CDCl_3$) of plakilactone G (**1**).
Figure S12. HMBC spectrum (500 MHz, $CDCl_3$) of plakilactone G (**1**).

Figure S13. ^{1}H NMR spectrum (600 MHz, $CDCl_3$) of plakilactone H (**2**).
Figure S14. COSY spectrum (600 MHz, $CDCl_3$) of plakilactone H (**2**).
Figure S15. HSQC spectrum (600 MHz, $CDCl_3$) of plakilactone H (**2**).
Figure S16. HMBC spectrum (600 MHz, $CDCl_3$) of plakilactone H (**2**).
Figure S17. Selective 1D-NOESY spectrum (600 MHz, $CDCl_3$) of Me16 of plakilactone H (**2**).
Figure S18. Selective 1D-NOESY spectrum (600 MHz, $CDCl_3$) of Me12 of plakilactone H (**2**).
Figure S19. Selective 1D-NOESY spectrum (600 MHz, $CDCl_3$) of H15a of plakilactone H (**2**).
Figure S20. Selective 1D-NOESY spectrum (600 MHz, $CDCl_3$) of H15b of plakilactone H (**2**).
Figure S21. Selective 1D-NOESY spectrum (600 MHz, $CDCl_3$) of H9 of plakilactone H (**2**).
Figure S22. Selective 1D-NOESY spectrum (600 MHz, $CDCl_3$) of H13a of plakilactone H (**2**).
Figure S23. Selective 1D-NOESY spectrum (600 MHz, $CDCl_3$) of H5b of plakilactone H (**2**).
Figure S24. Selective 1D-NOESY spectrum (600 MHz, $CDCl_3$) of H11 of plakilactone H (**2**).
Figure S25. Selective 1D-NOESY spectrum (600 MHz, $CDCl_3$) of H7 of plakilactone H (**2**).
Figure S26. Selective 1D-NOESY spectrum (600 MHz, $CDCl_3$) of H8 of plakilactone H (**2**).
Figure S27. Selective 1D-NOESY spectrum (600 MHz, $CDCl_3$) of H3 of plakilactone H (**2**).
Table S2. Experimental and calculated ^{13}C chemical shifts values for **2a,b,e,f**.
Table S3. Experimental and calculated ^{13}C chemical shifts values for **1a-d**.
Figure S28. Selective 1D-NOESY spectrum (500 MHz, $CDCl_3$) of Me14 of plakilactone G (**1**).
Figure S29. Selective 1D-NOESY spectrum (500 MHz, $CDCl_3$) of Me16 of plakilactone G (**1**).
Figure S30. Selective 1D-NOESY spectrum (500 MHz, $CDCl_3$) of H9b of plakilactone G (**1**).
Figure S31. Selective 1D-NOESY spectrum (500 MHz, $CDCl_3$) of H13b of plakilactone G (**1**).
Figure S32. Selective 1D-NOESY spectrum (500 MHz, $CDCl_3$) of H5b of plakilactone G (**1**).
Figure S33. Selective 1D-NOESY spectrum (500 MHz, $CDCl_3$) of H11 of plakilactone G (**1**).
Figure S34. Selective 1D-NOESY spectrum (500 MHz, $CDCl_3$) of H3 of plakilactone G (**1**).
Table S4. Comparison of calculated and experimental inter-proton distances for plakilactone G (**1**).

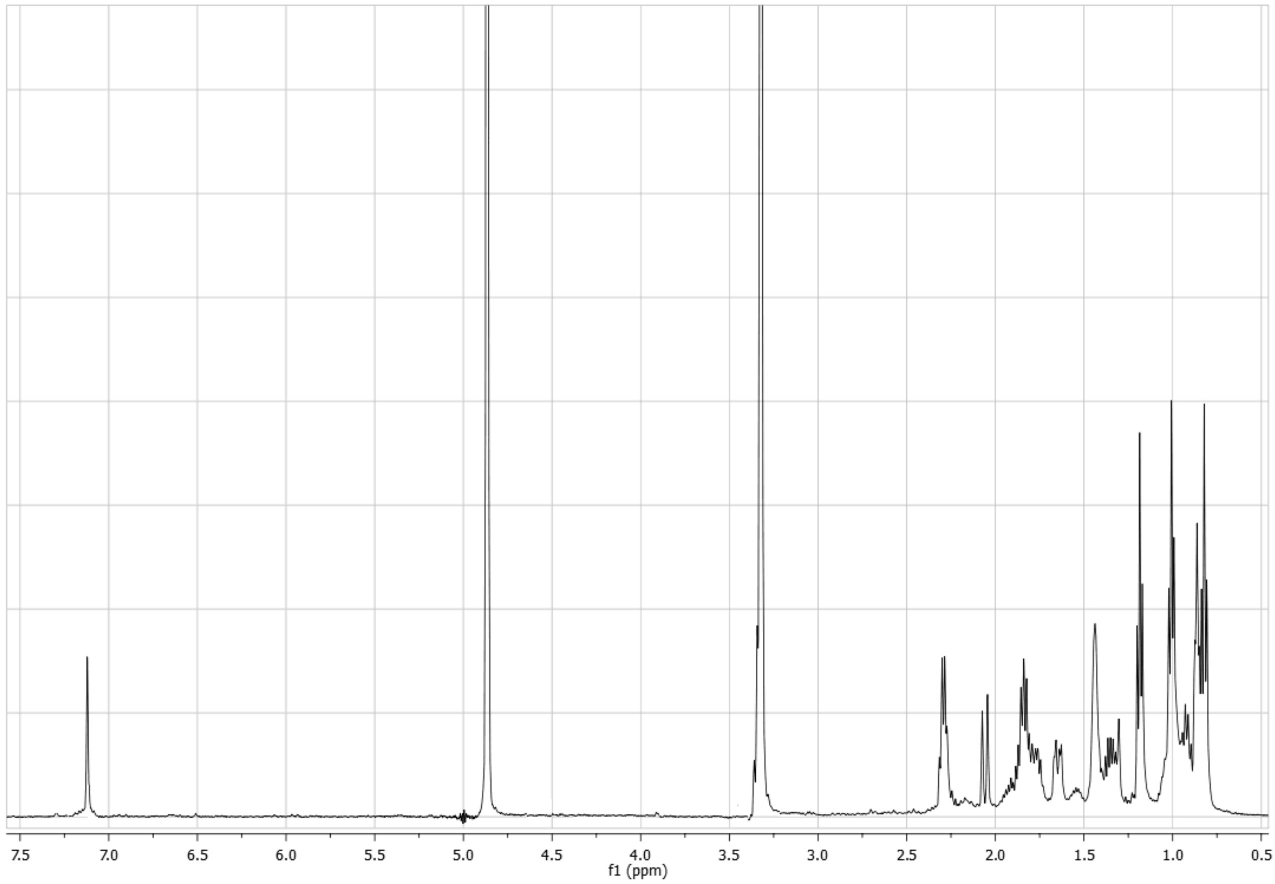

Figure S1: ^{1}H NMR spectrum (500 MHz, CD_3OD) of plakilactone G (**1**).

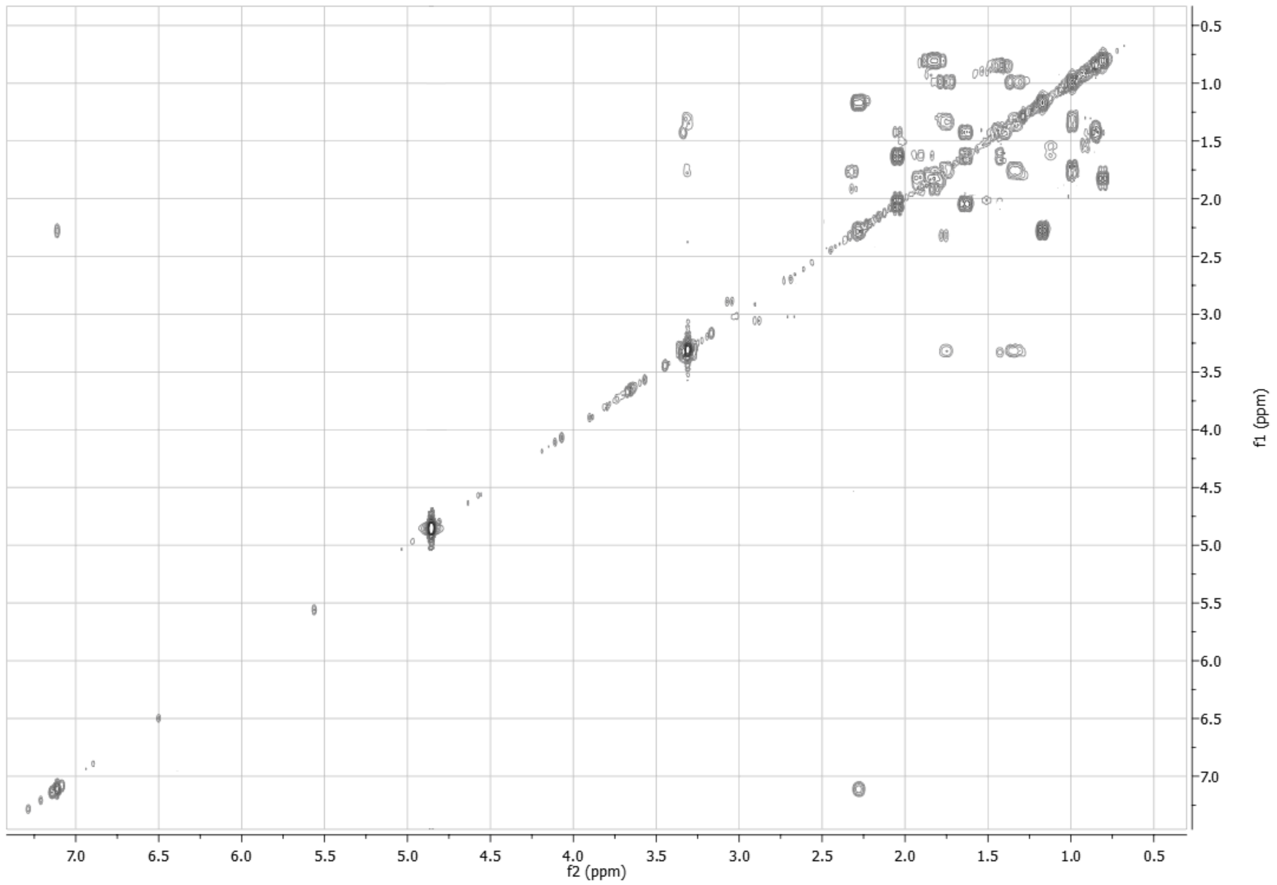

Figure S2: COSY spectrum (500 MHz, CD_3OD) of plakilactone G (**1**).

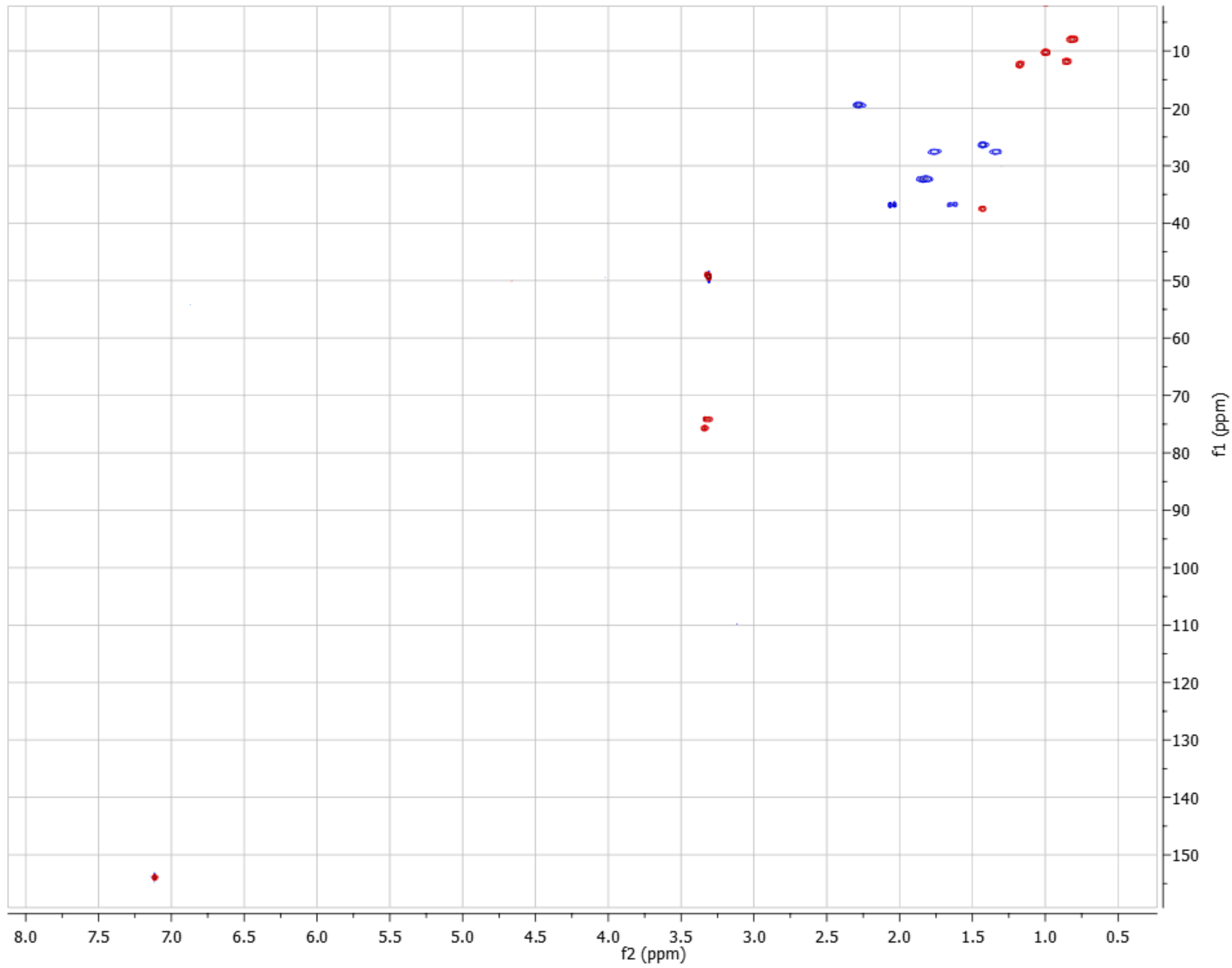

Figure S3: HSQC spectrum (500 MHz, CD_3OD) of plakilactone G (**1**).

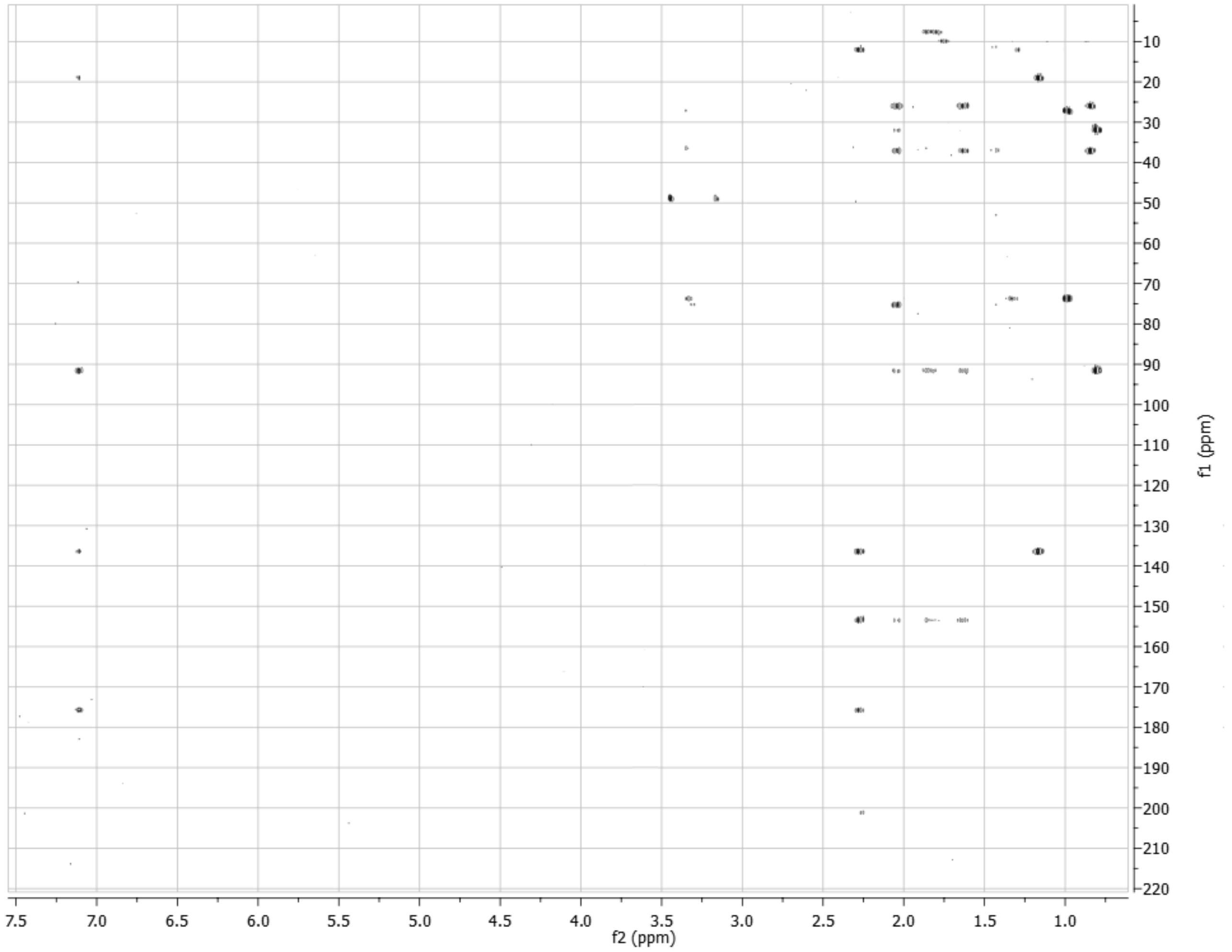

Figure S4: HMBC spectrum (500 MHz, CD_3OD) of plakilactone G (**1**).

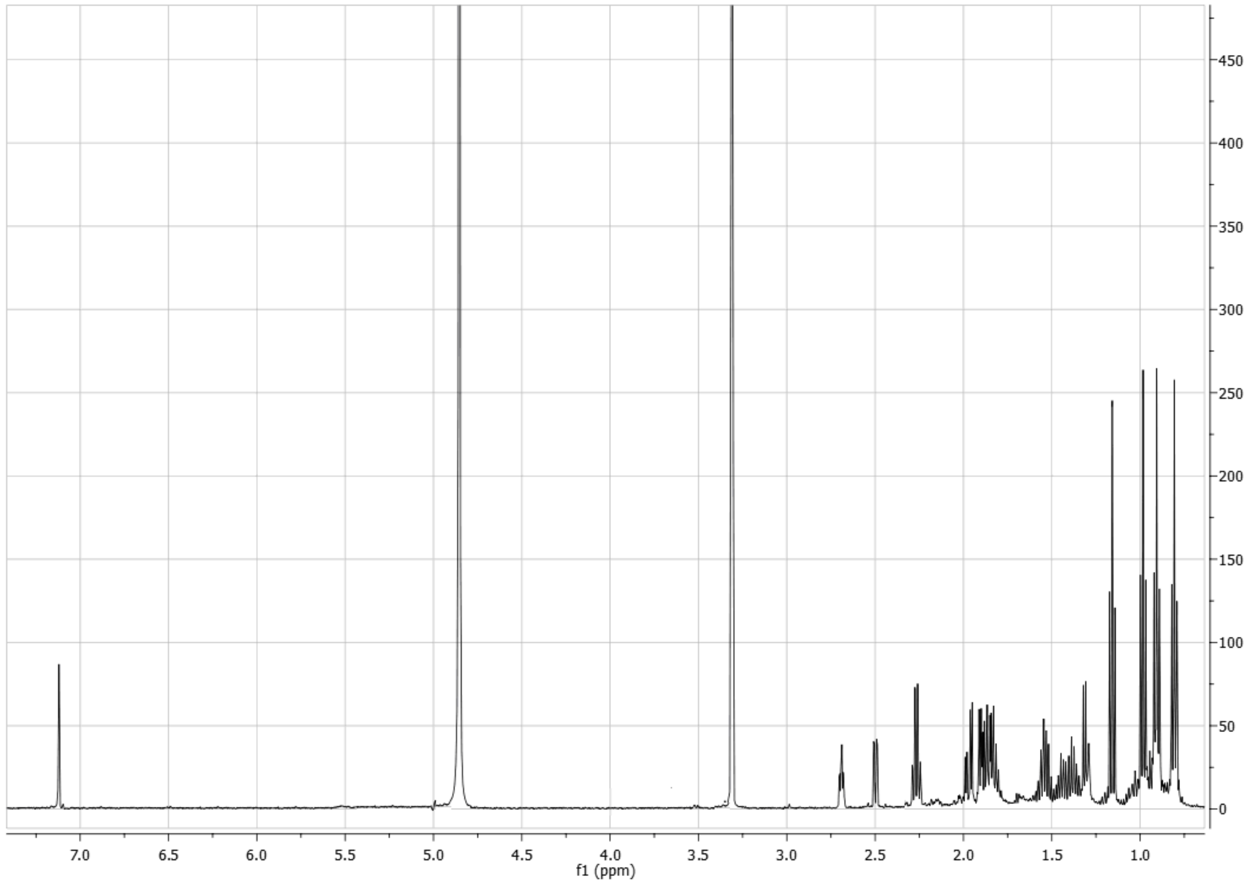

Figure S5: ^{1}H NMR spectrum (500 MHz, CD_3OD) of plakilactone H (**2**).

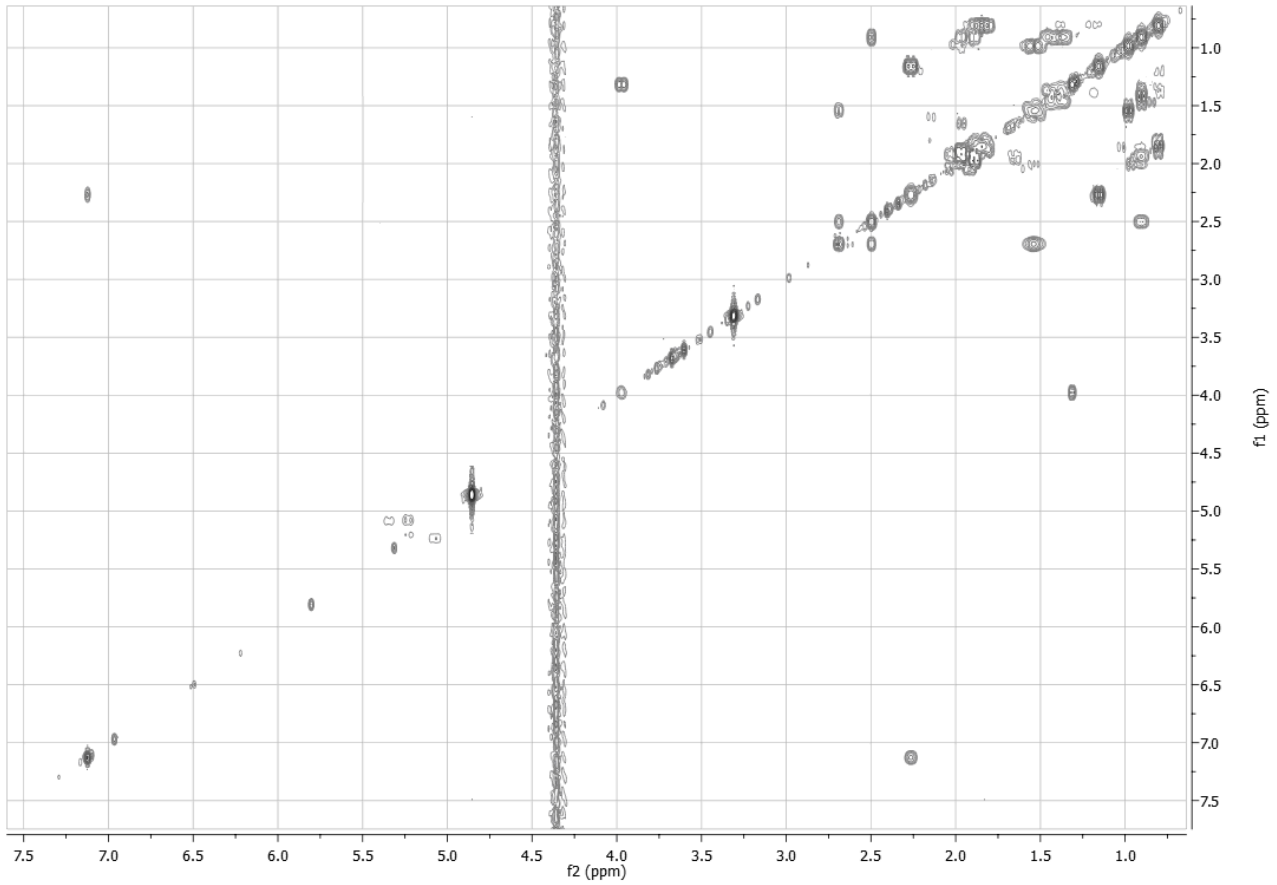

Figure S6: COSY spectrum (500 MHz, CD_3OD) of plakilactone H (**2**).

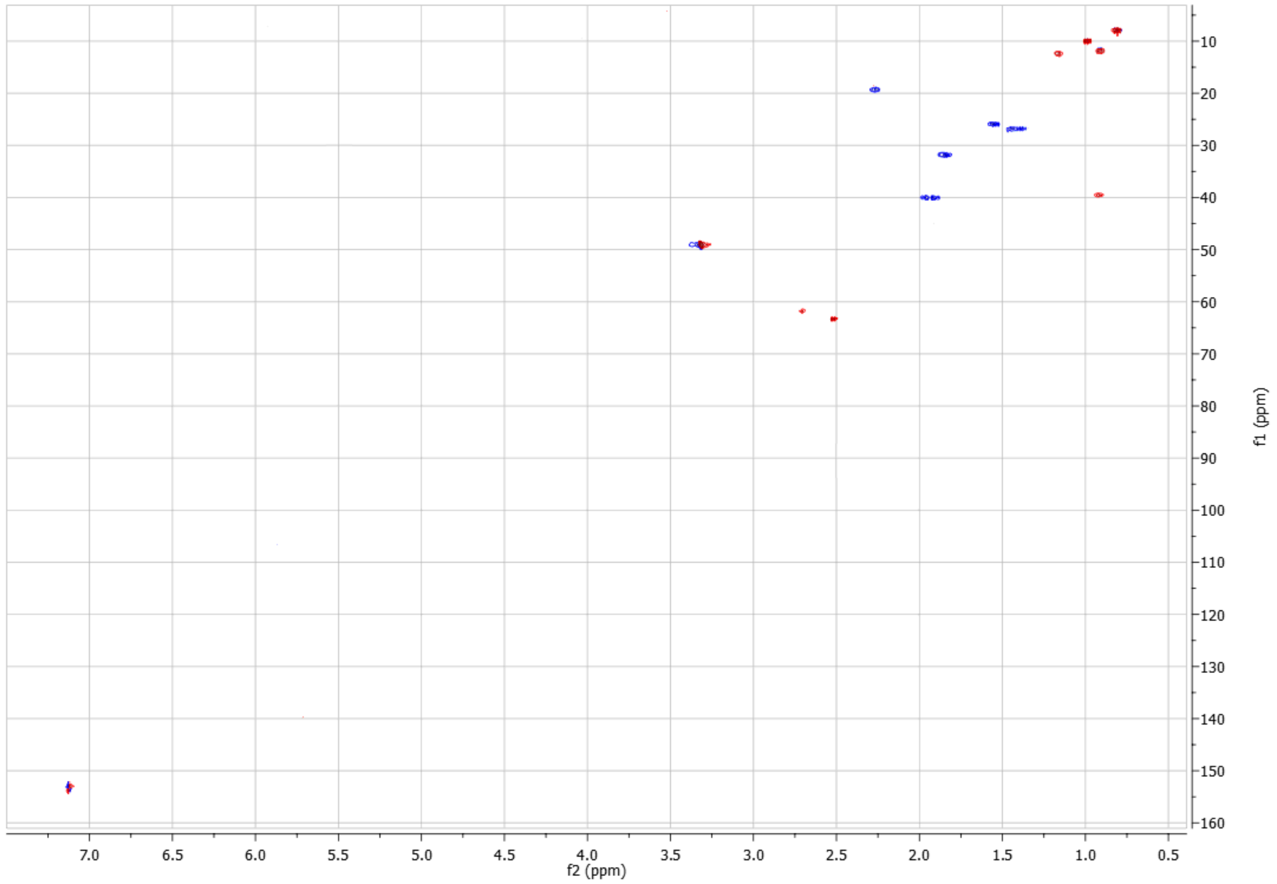

Figure S7: HSQC spectrum (500 MHz, CD_3OD) of plakilactone H (**2**).

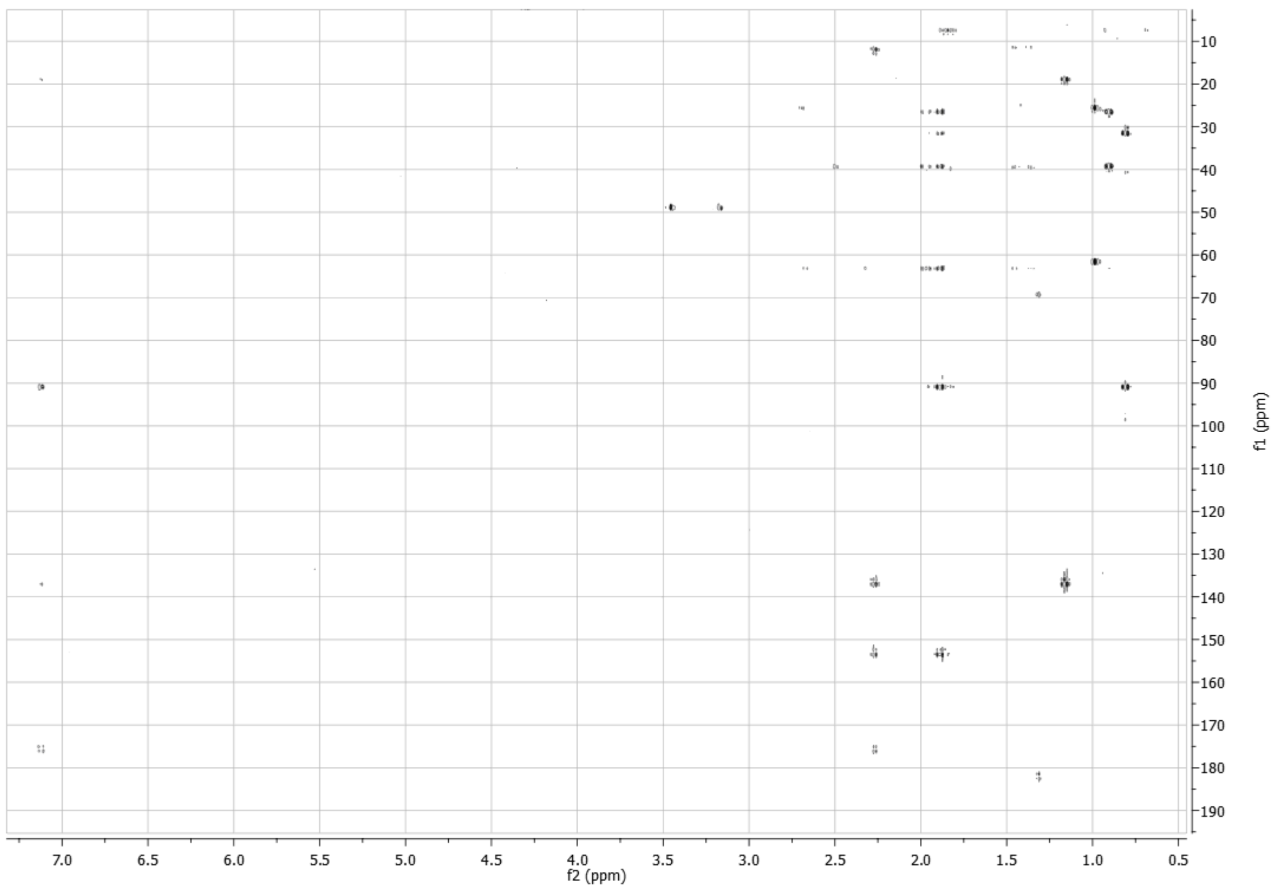

Figure S8: HMBC spectrum (500 MHz, CD_3OD) of plakilactone H (**2**).

Table S1: NMR Data of plakilactones G (**1**, 500 MHz, $CDCl_3$) and H (**2**, 600 MHz, $CDCl_3$).

	**1**		**2**	
position	δ_H	δ_C	δ_H	δ_C
1	-	173.1	-	173.6
2	-	135.5	-	136.1
3	6.87	151.0	6.86	150.2
4	-		-	89.1
5	1.71<br>2.05	36.2	1.87<br>1.97	39.1
6	1.37	35.6	0.84	38.2
7	3.46	75.5	2.43	62.1
8	3.49	73.6	2.62	60.9
9	1.38<br>1.52	24.0	1.54	24.9
10	0.99	10.1	0.98	9.7
11	2.32	18.5	1.14	18.3
12	1.15	12.1	2.29	11.8
13	1.72<br>1.86	30.9	1.76<br>1.86	31.2
14	0.81	7.9	0.83	7.73
15	1.35	25.3	1.34<br>1.46	25.8
16	0.89	10.8	0.88	11.4

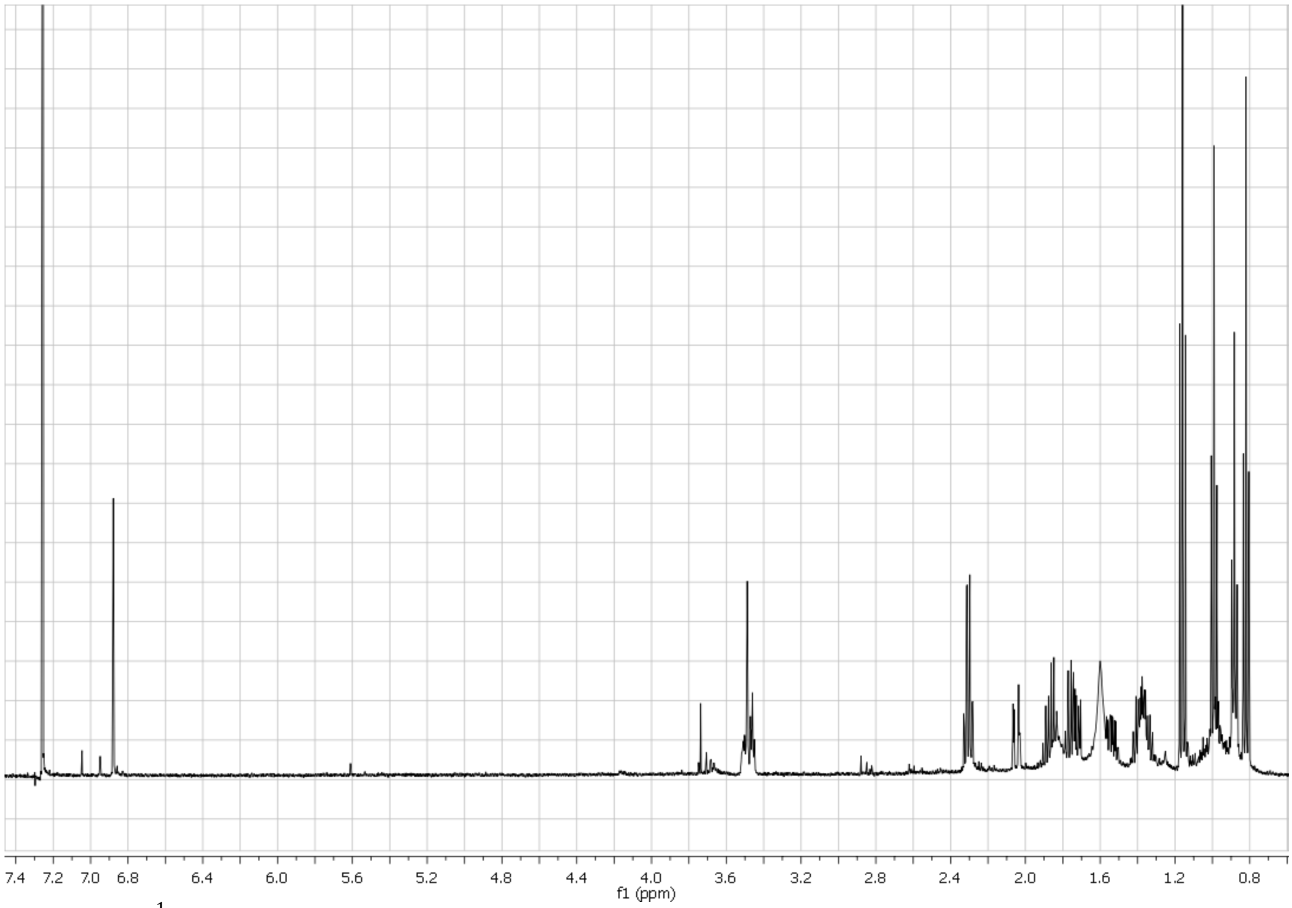

Figure S9: ^{1}H NMR spectrum (500 MHz, CDCl3) of plakilactone G (**1**).

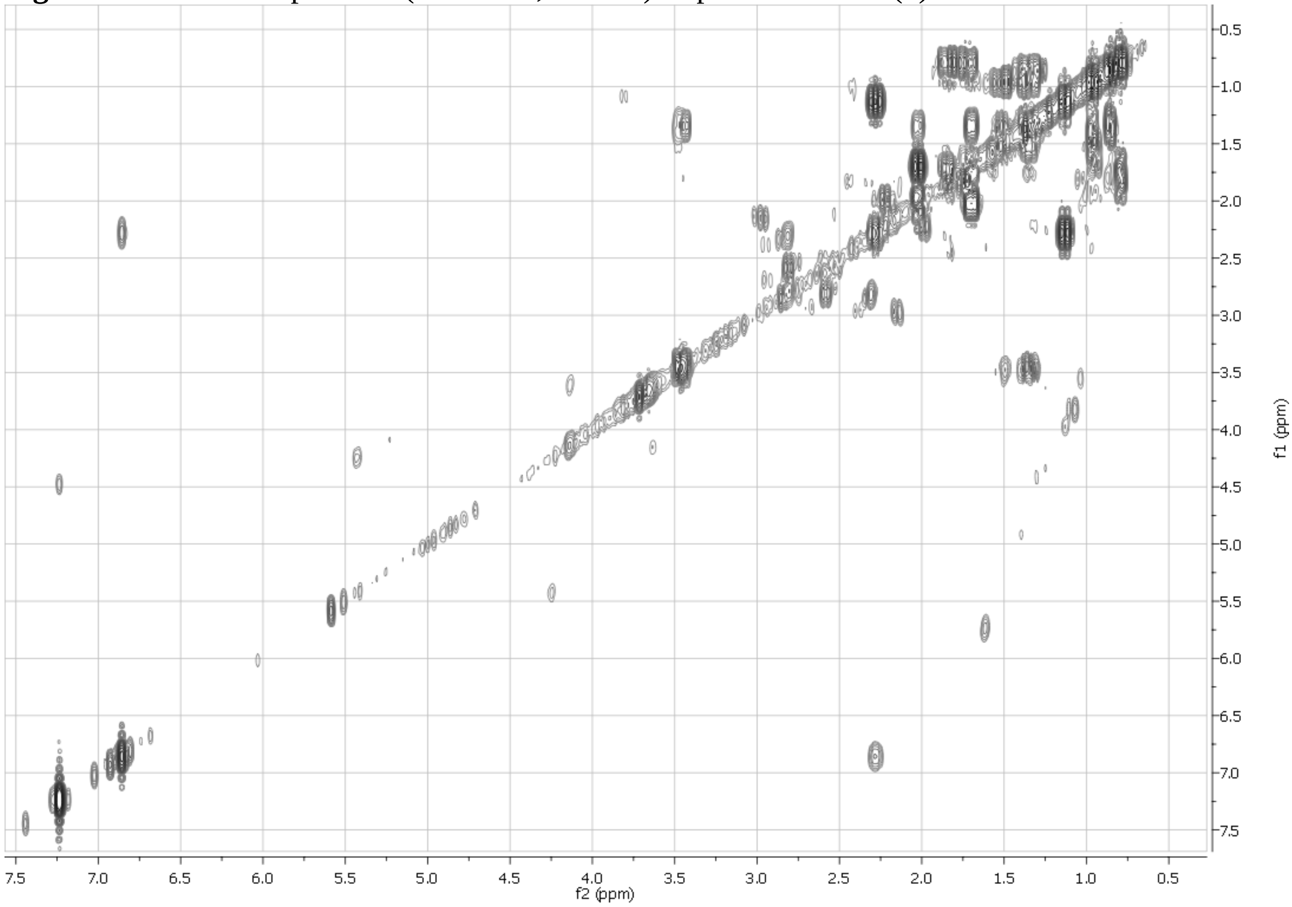

Figure S10: COSY spectrum (500 MHz, CDCl3) of plakilactone G (**1**).

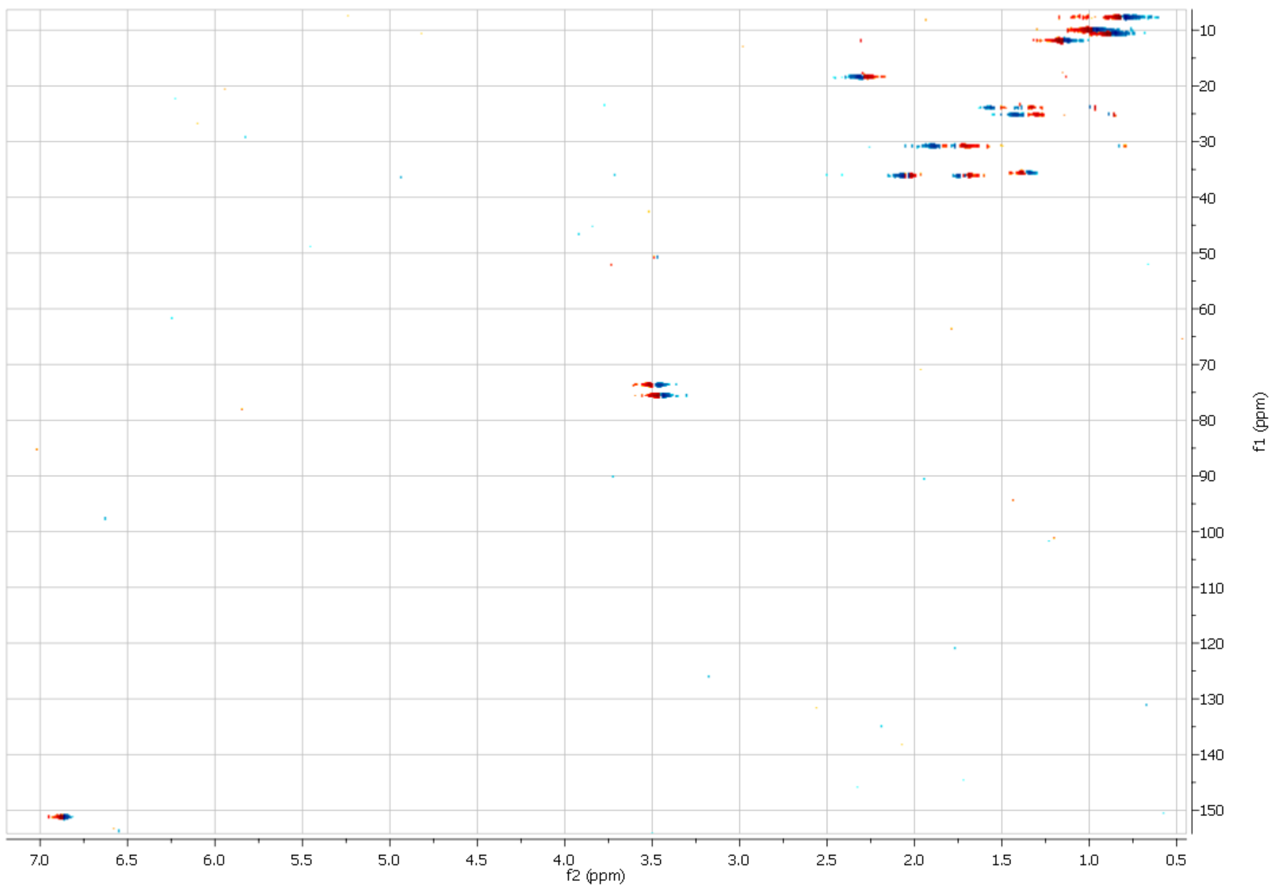

Figure S11: HSQC spectrum (500 MHz, $CDCl_3$) of plakilactone G (**1**).

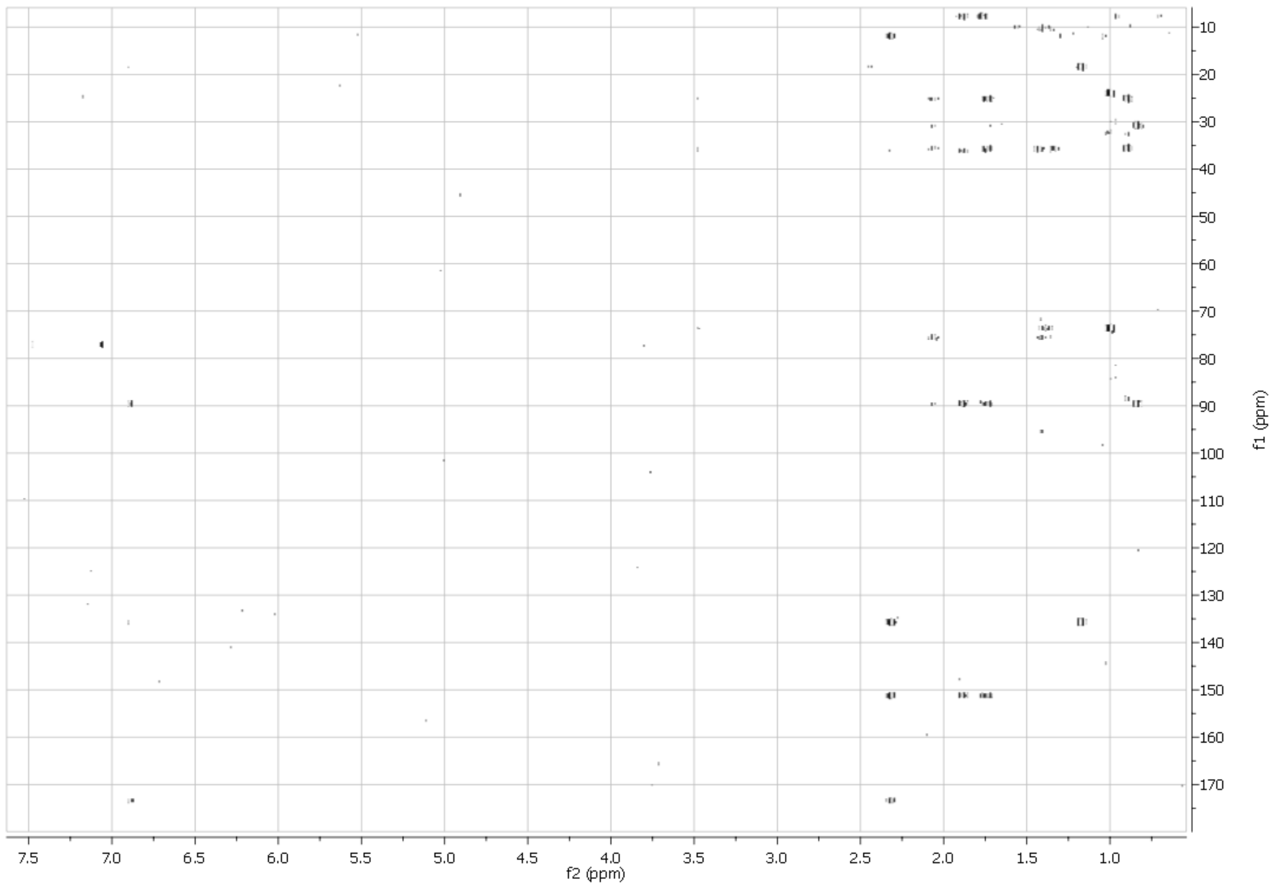

Figure S12: HMBC spectrum (500 MHz, $CDCl_3$) of plakilactone G (**1**).

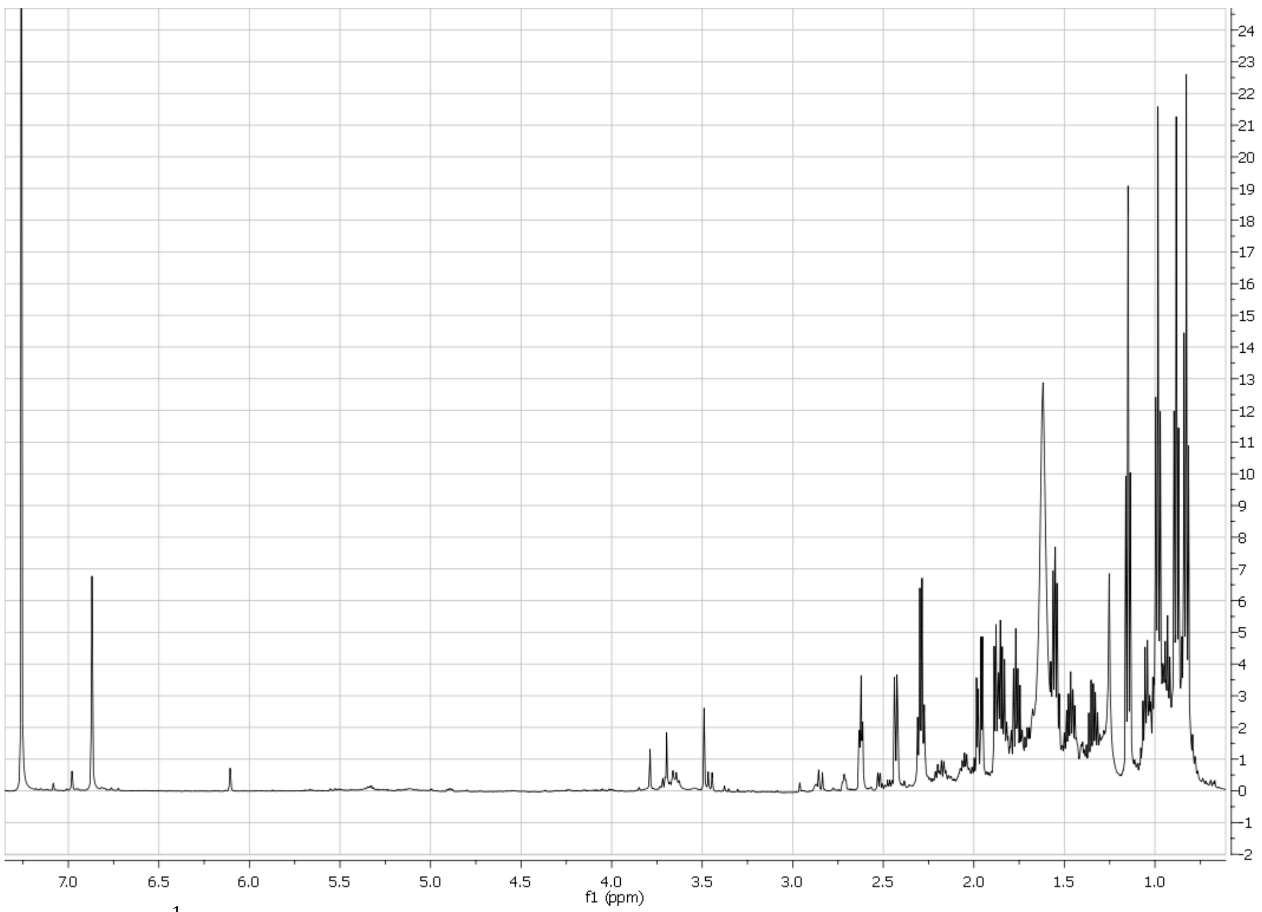

Figure S13: ^{1}H NMR spectrum (600 MHz, $CDCl_3$) of plakilactone H (**2**).

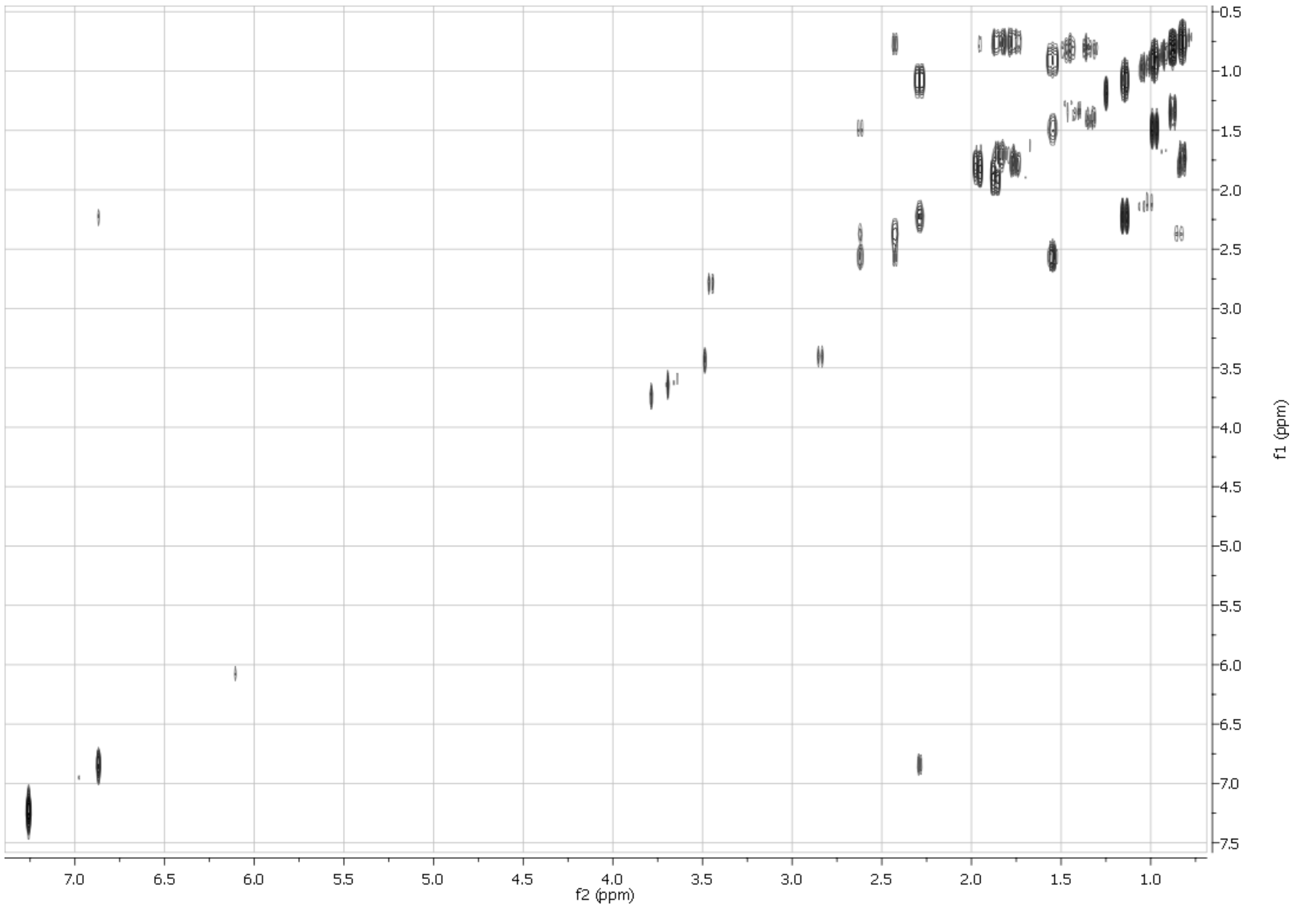

Figure S14: COSY spectrum (600 MHz, $CDCl_3$) of plakilactone H (**2**).

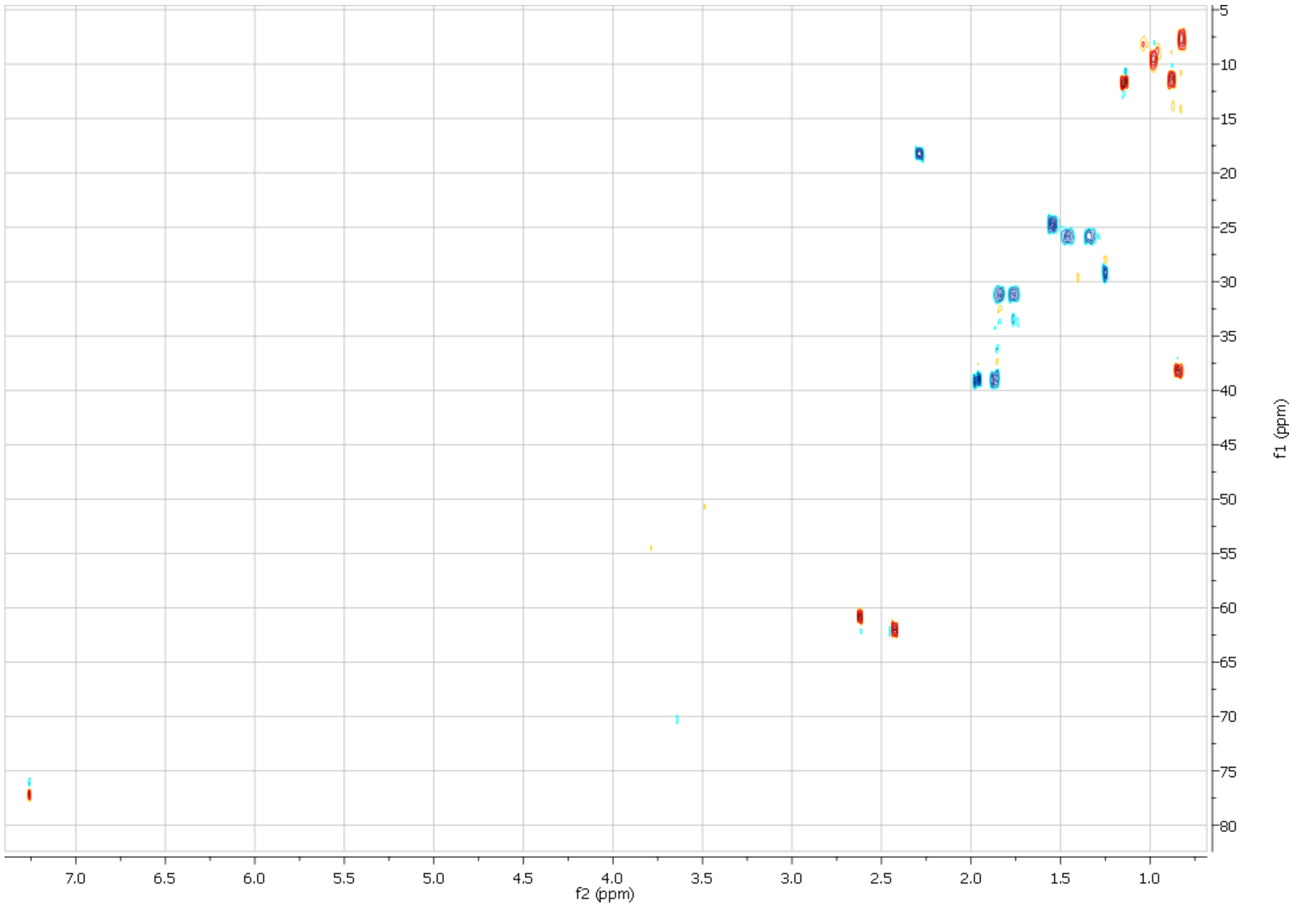

Figure S15: HSQC spectrum (600 MHz, $CDCl_3$) of plakilactone H (**2**).

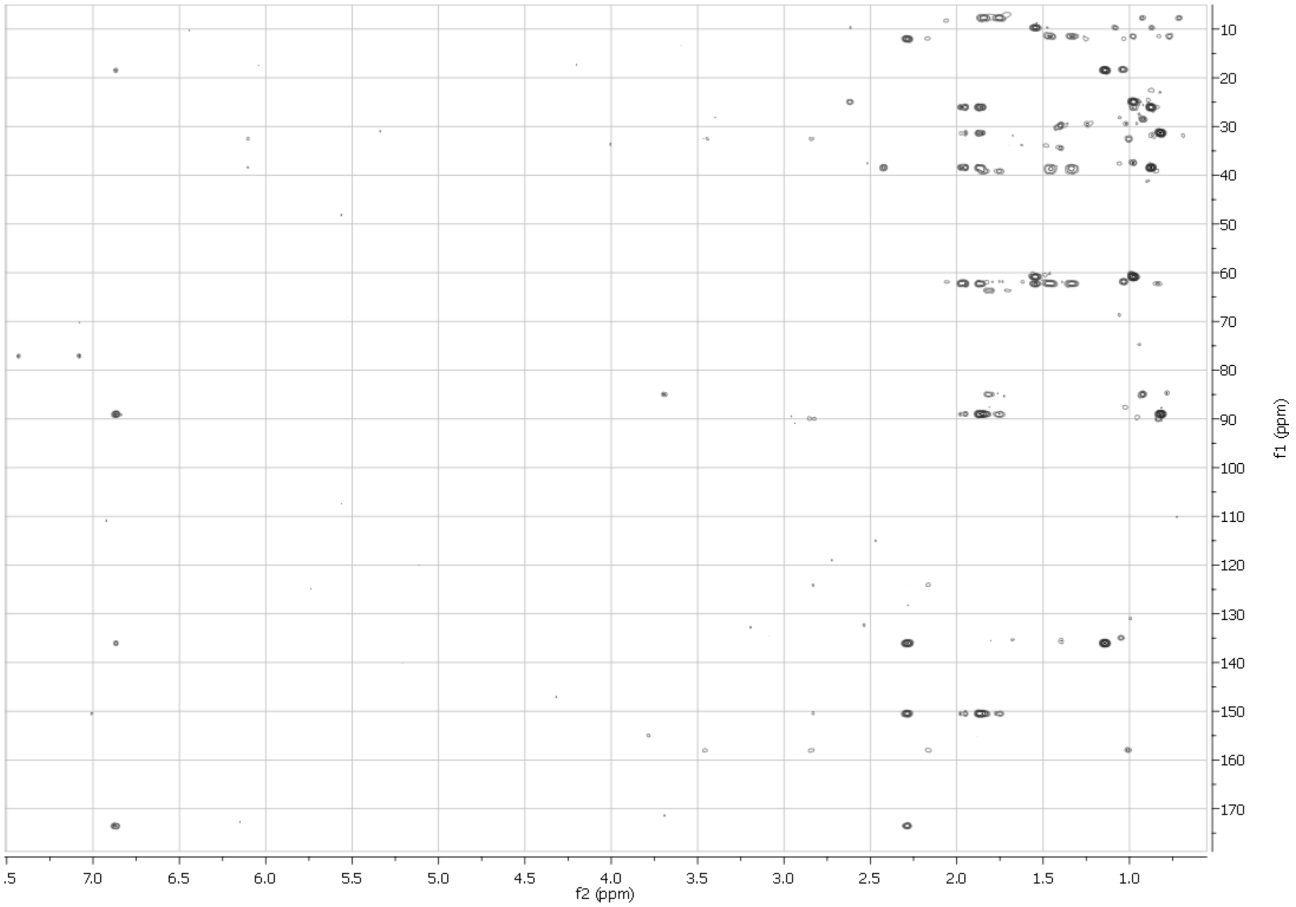

Figure S16: HMBC spectrum (600 MHz, $CDCl_3$) of plakilactone H (**2**).

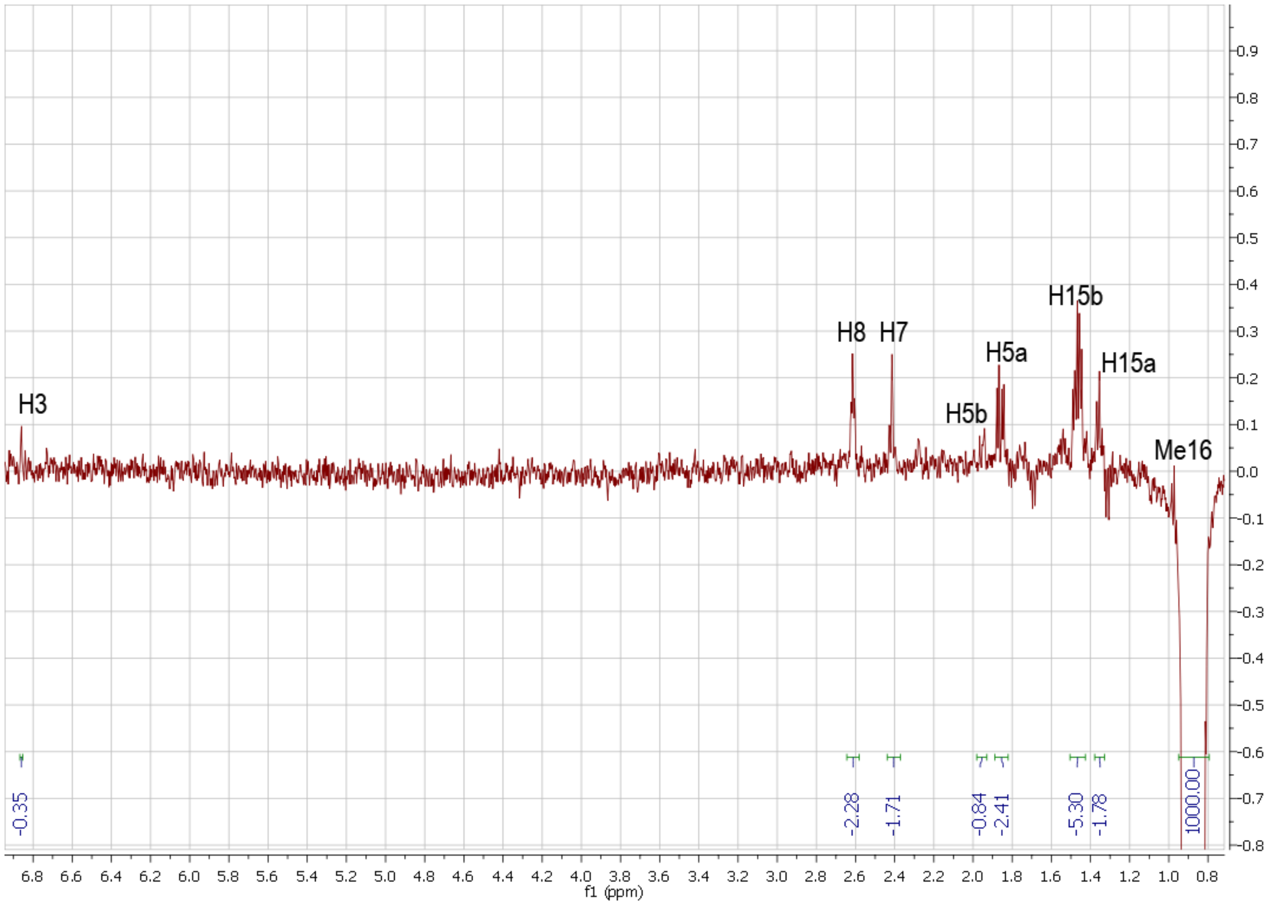

Figure S17: Selective 1D-NOESY spectrum (600 MHz, $CDCl_3$) of Me16 of plakilactone H (**2**).

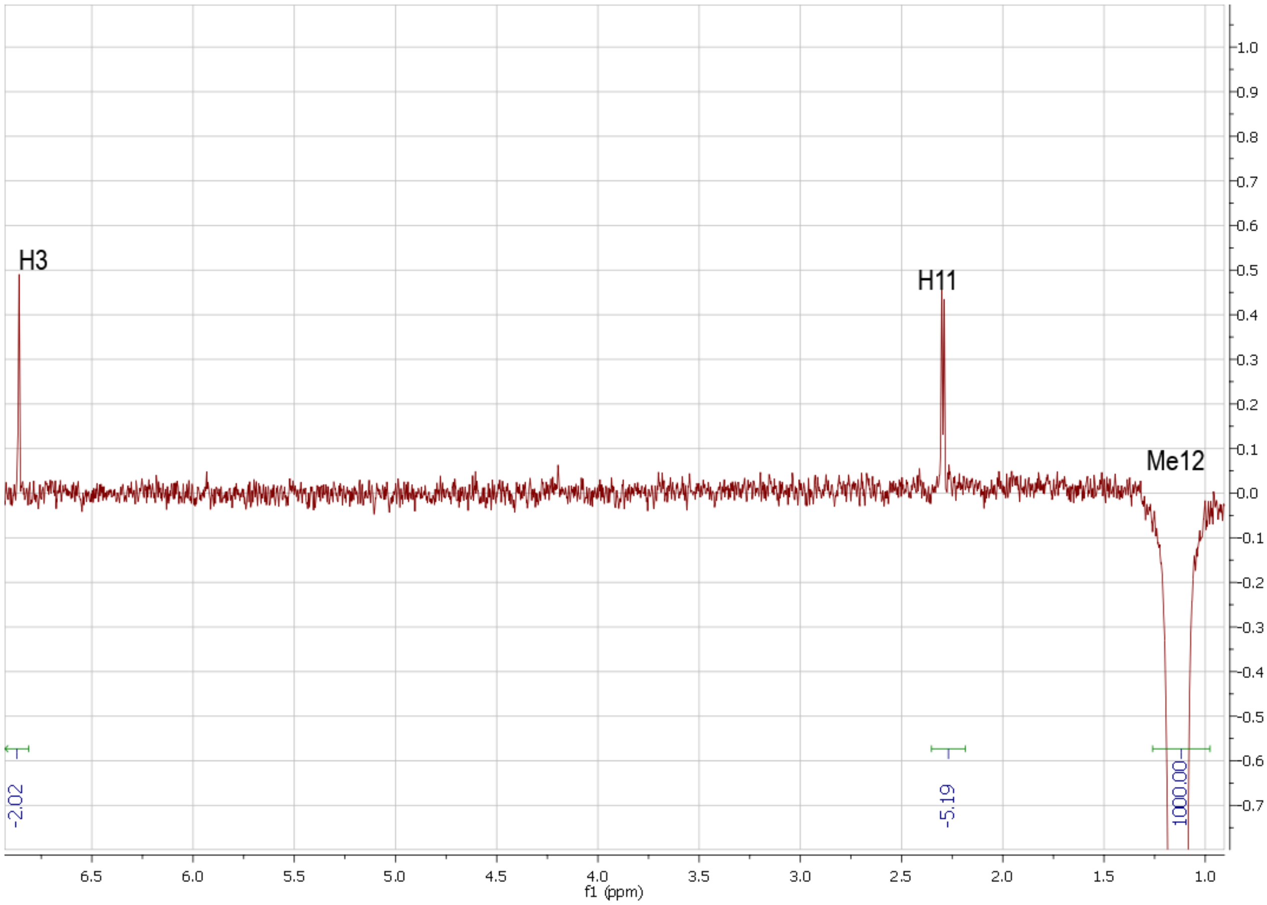

Figure S18: Selective 1D-NOESY spectrum (600 MHz, $CDCl_3$) of Me12 of plakilactone H (**2**).

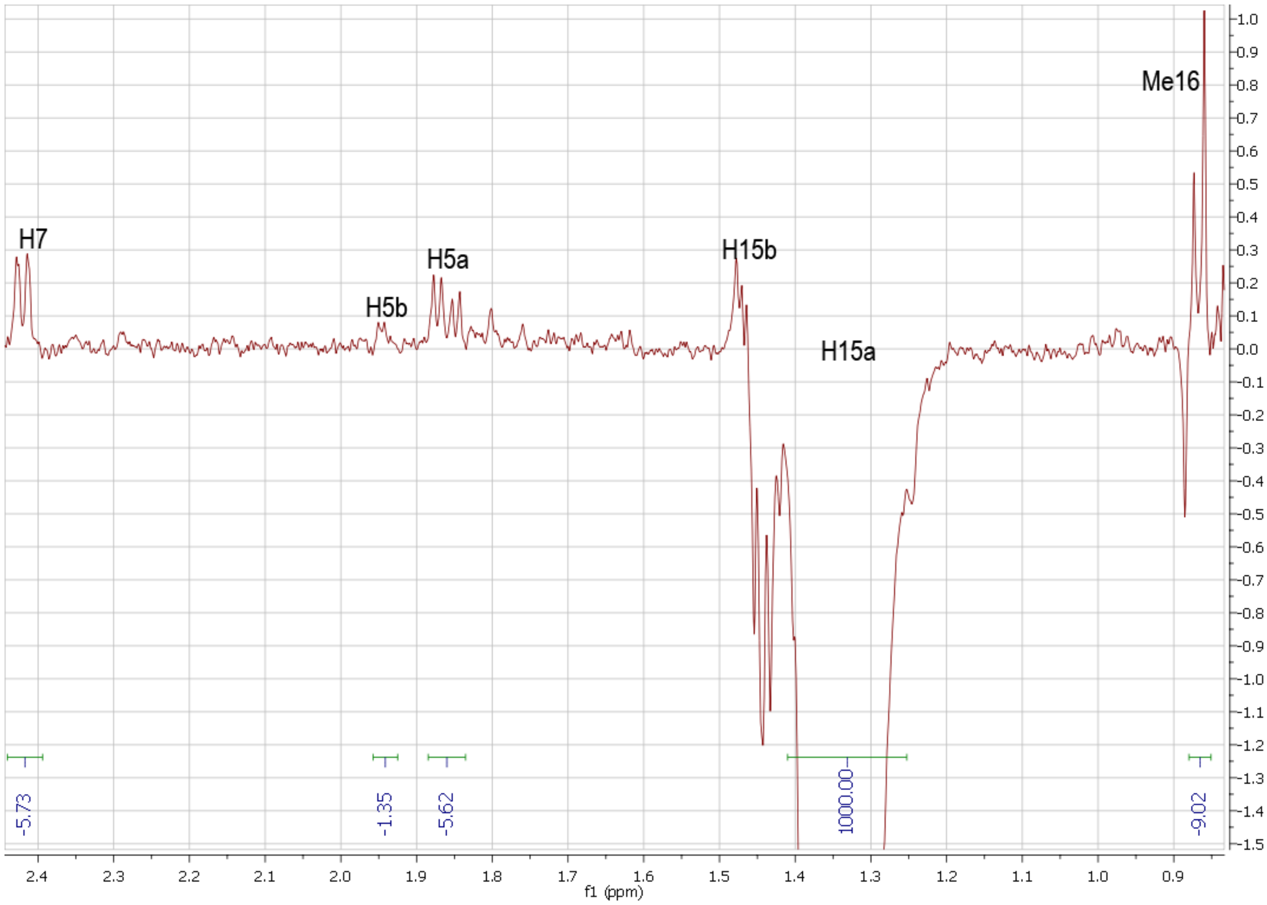

Figure S19: Selective 1D-NOESY spectrum (600 MHz, $CDCl_3$) of H15a of plakilactone H (**2**).

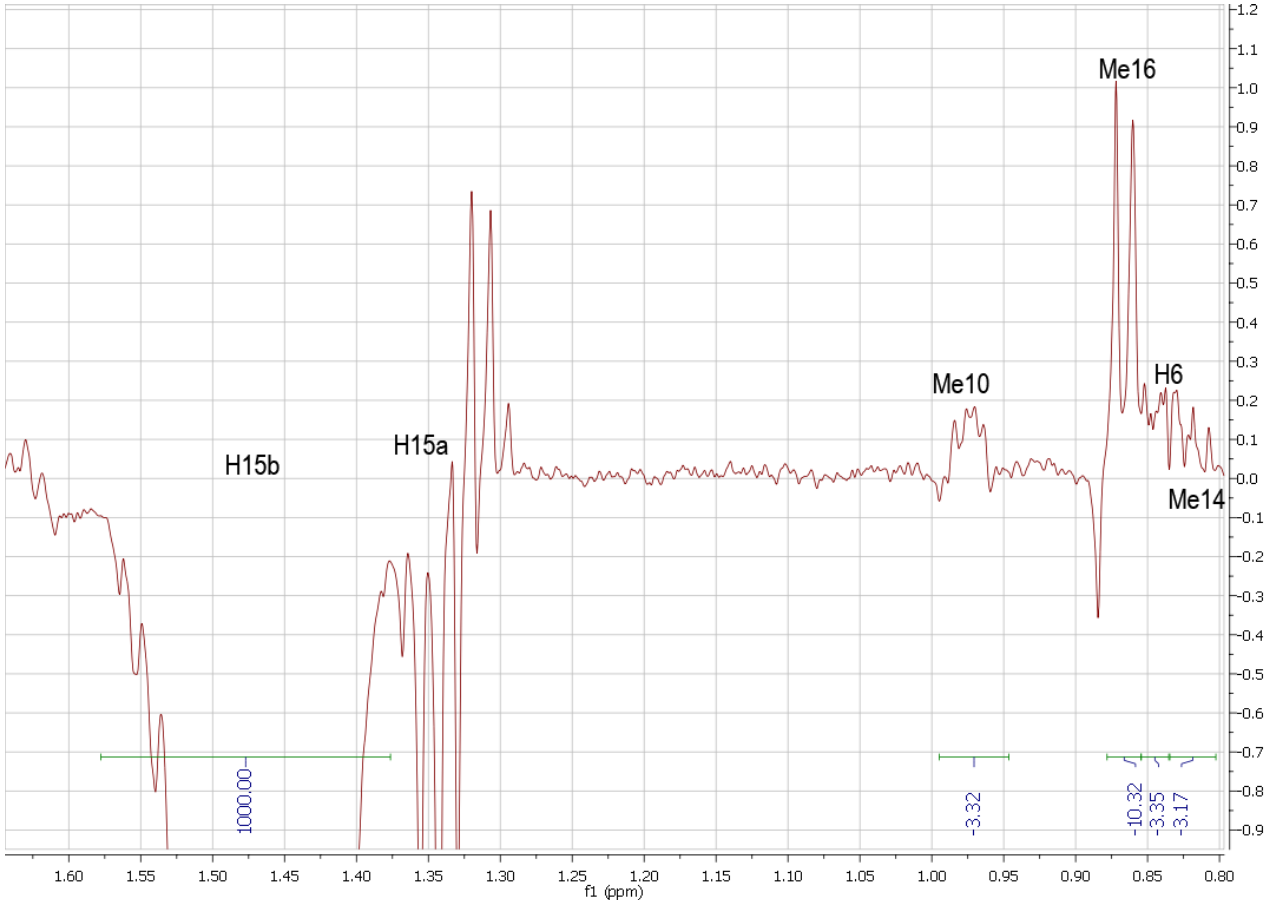

Figure S20: Selective 1D-NOESY spectrum (600 MHz, $CDCl_3$) of H15b of plakilactone H (**2**).

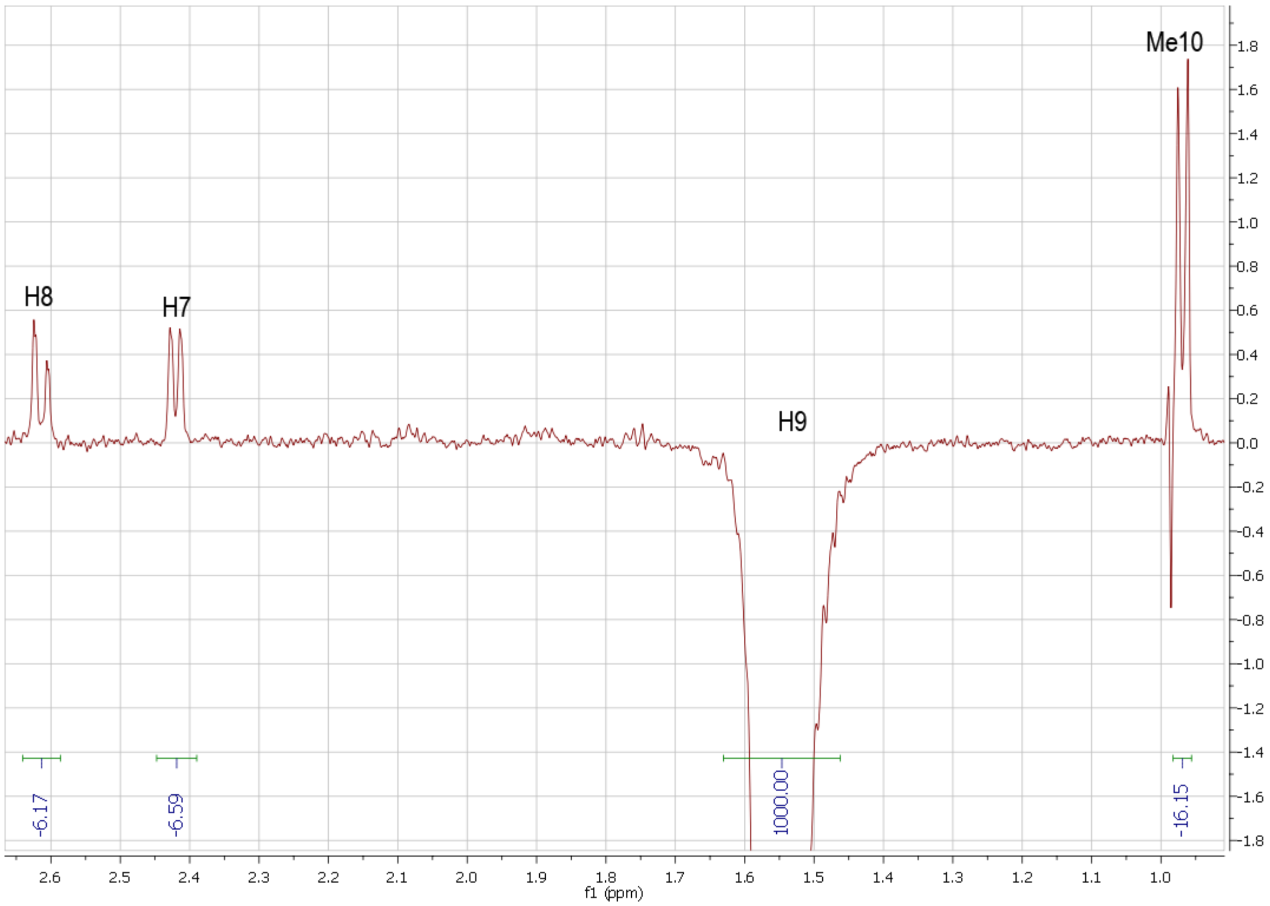

Figure S21: Selective 1D-NOESY spectrum (600 MHz, $CDCl_3$) of H9 of plakilactone H (**2**).

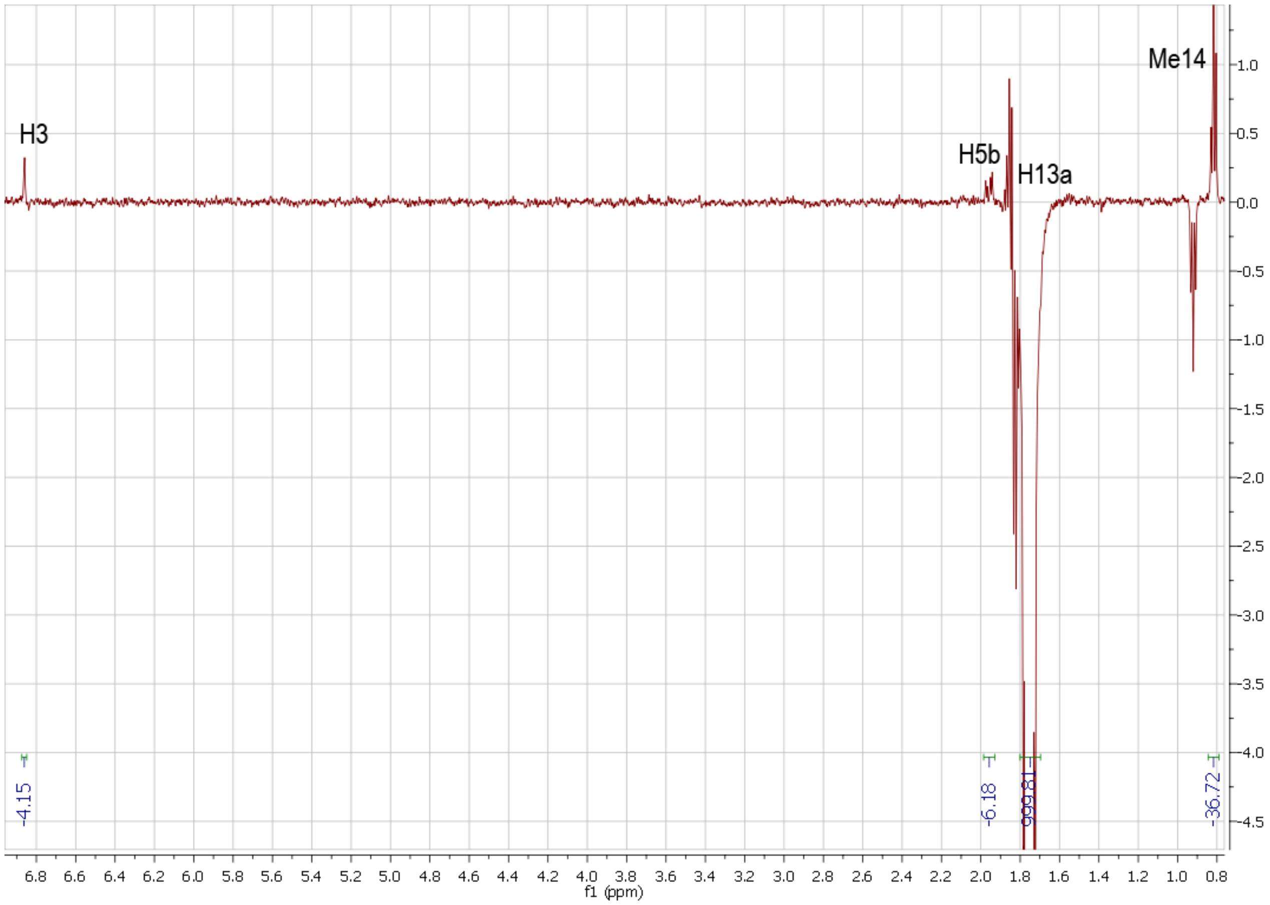

Figure S22: Selective 1D-NOESY spectrum (600 MHz, $CDCl_3$) of H13a of plakilactone H (**2**).

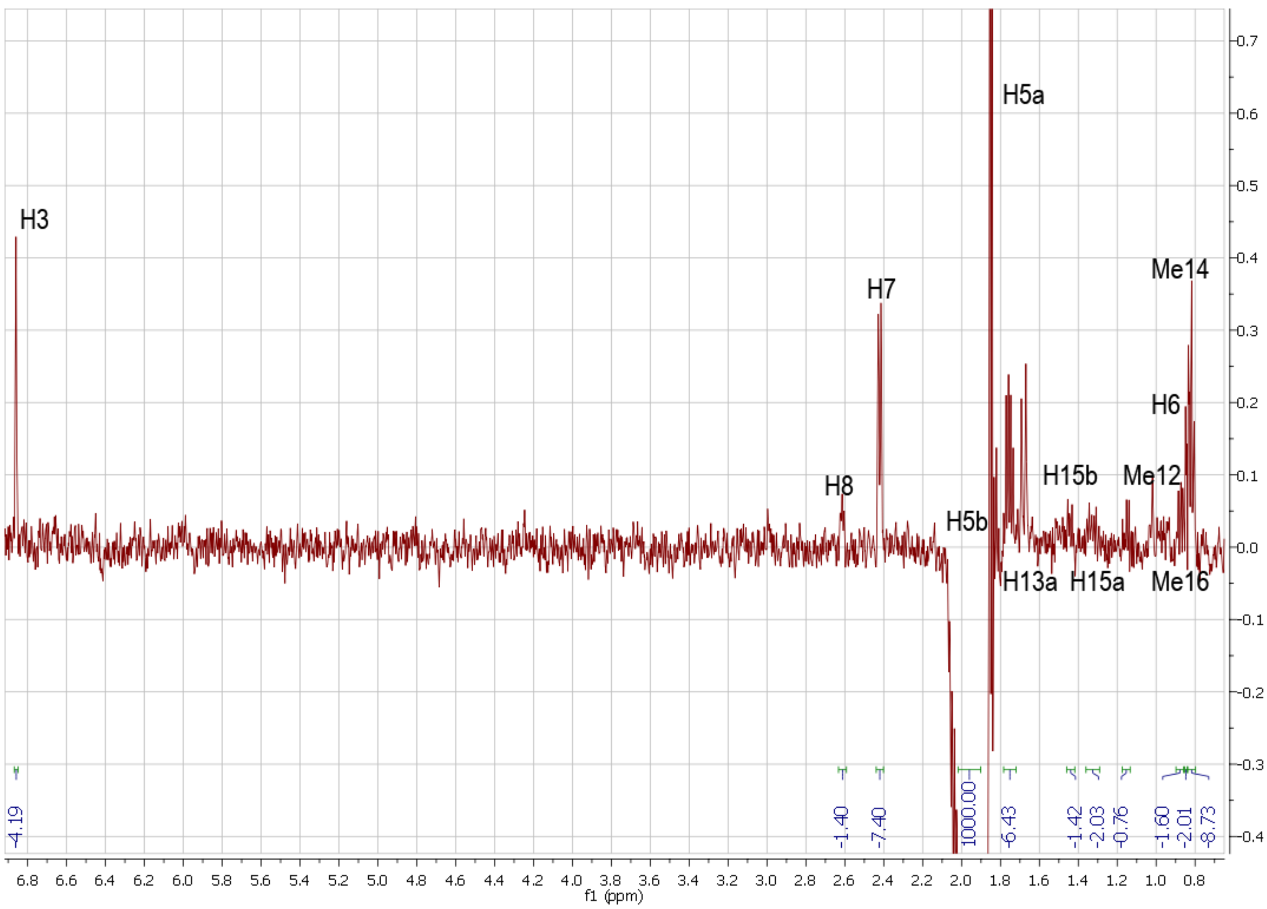

Figure S23: Selective 1D-NOESY spectrum (600 MHz, $CDCl_3$) of H5b of plakilactone H (**2**).

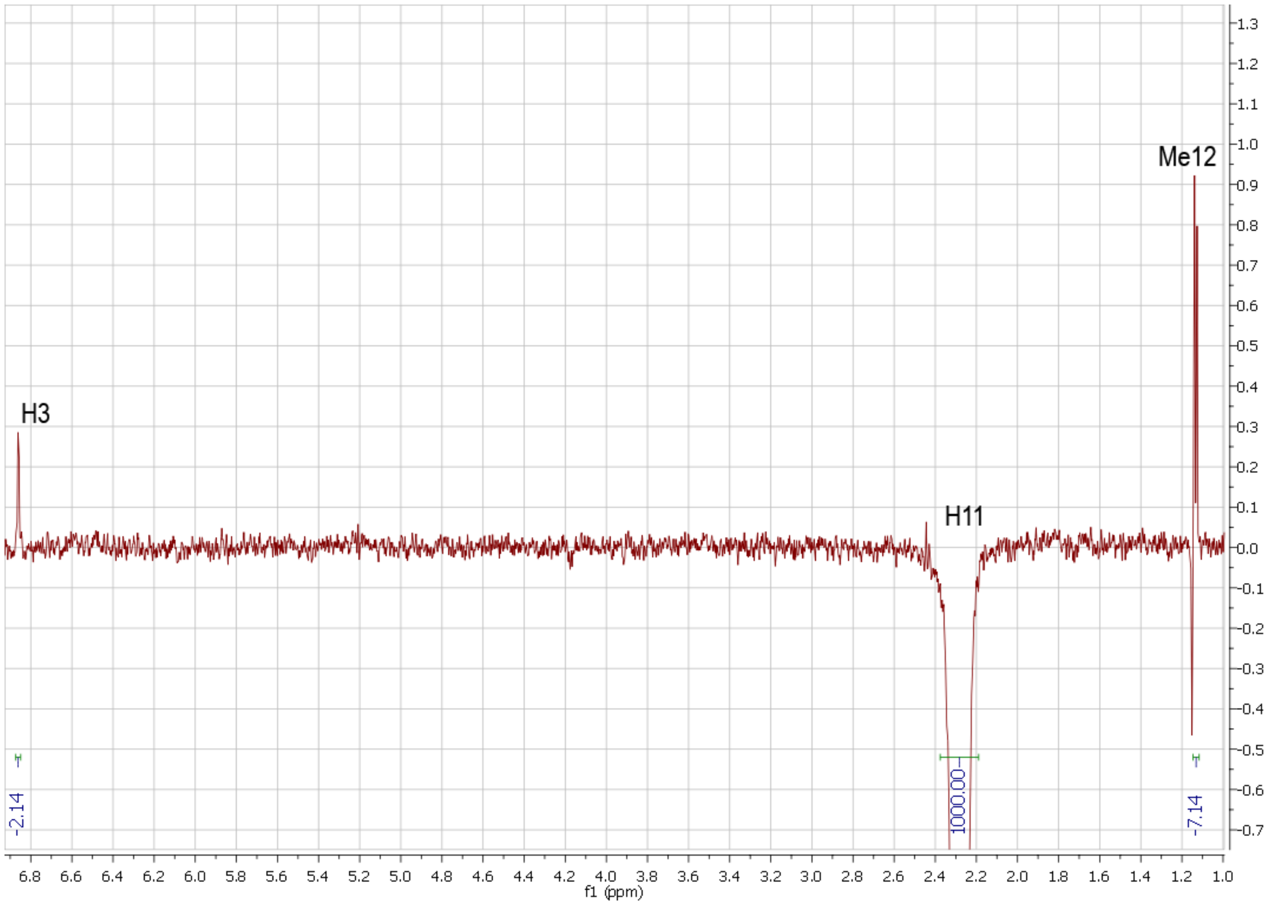

Figure S24: Selective 1D-NOESY spectrum (600 MHz, $CDCl_3$) of H11 of plakilactone H (**2**).

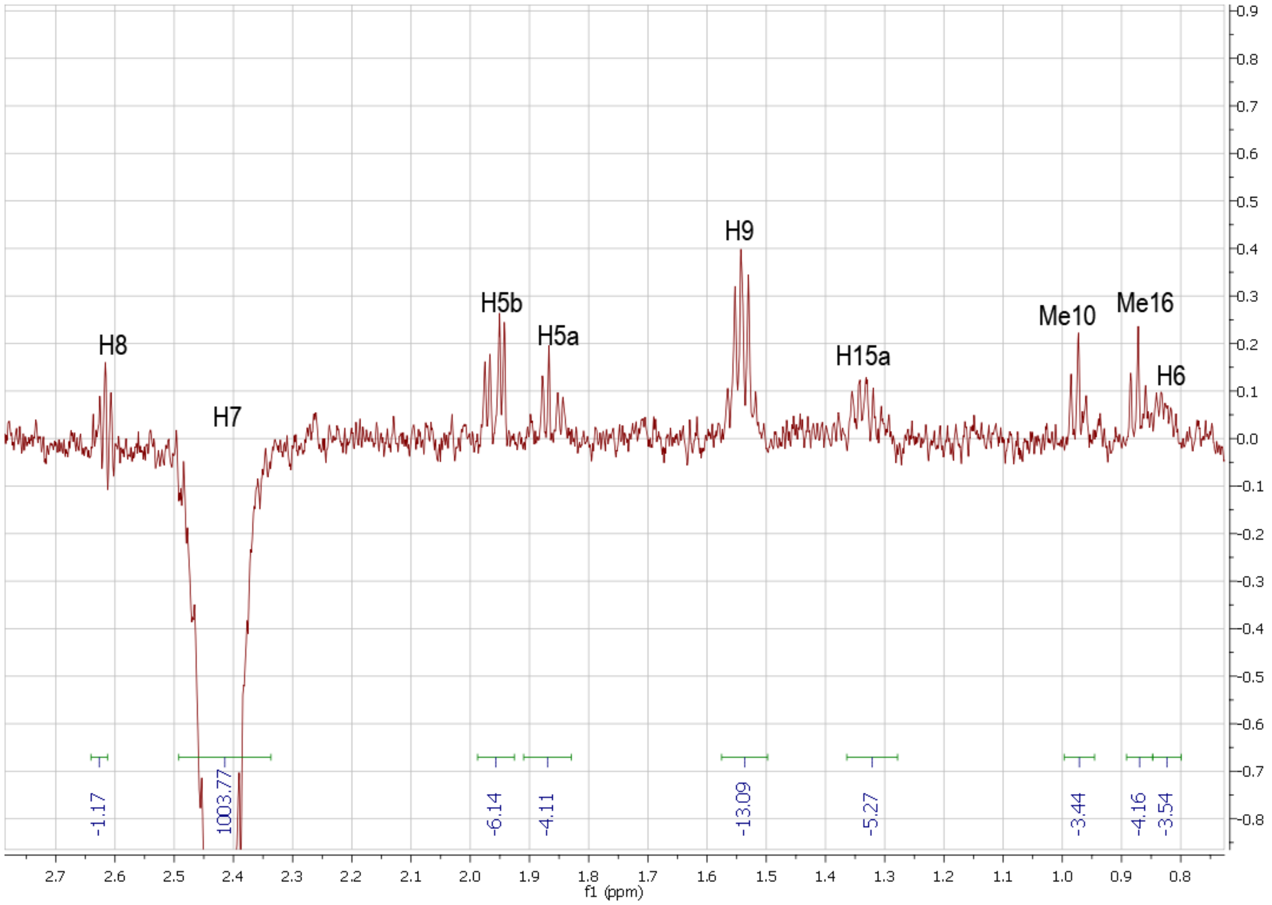

Figure S25: Selective 1D-NOESY spectrum (600 MHz, $CDCl_3$) of H7 of plakilactone H (**2**).

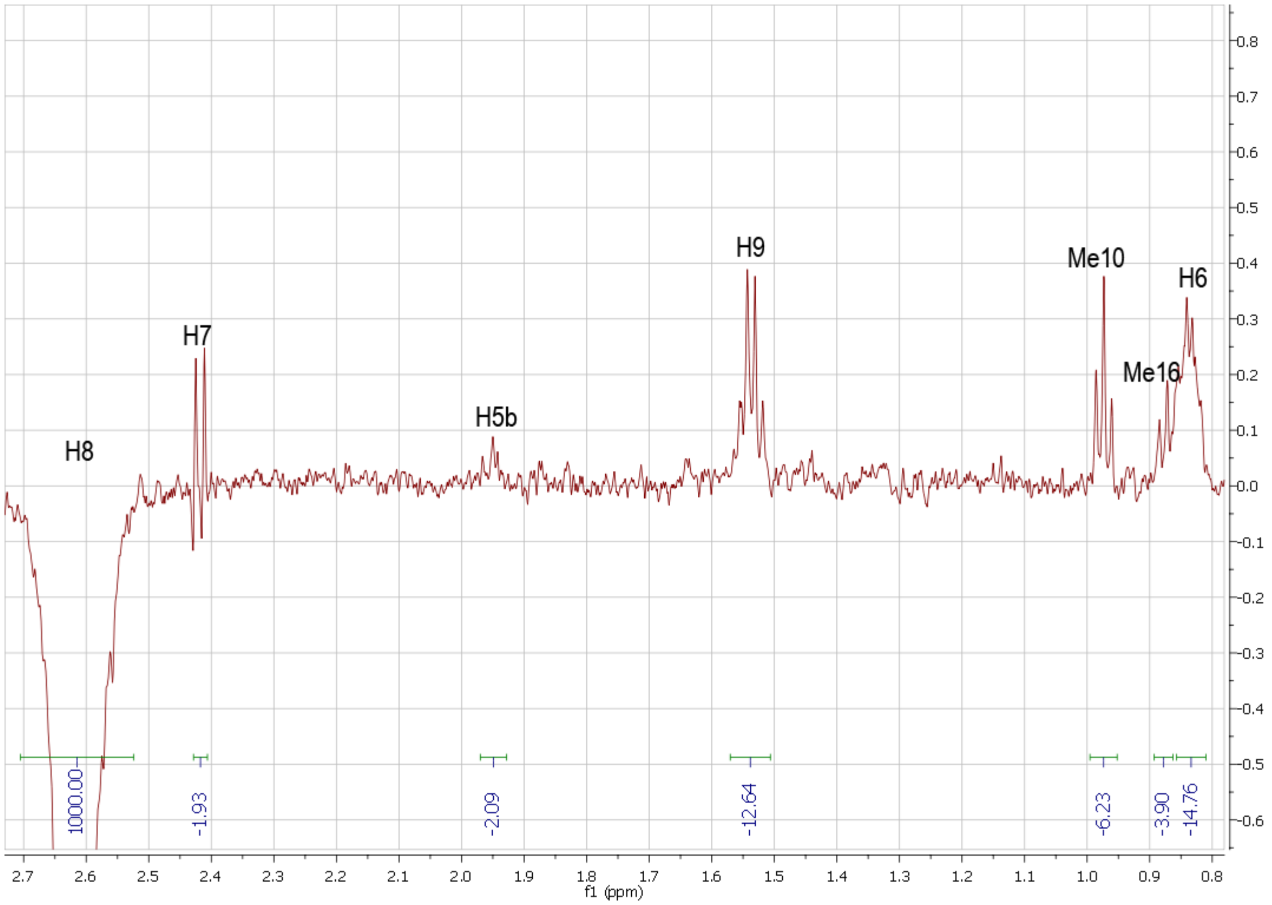

Figure S26: Selective 1D-NOESY spectrum (600 MHz, $CDCl_3$) of H8 of plakilactone H (**2**).

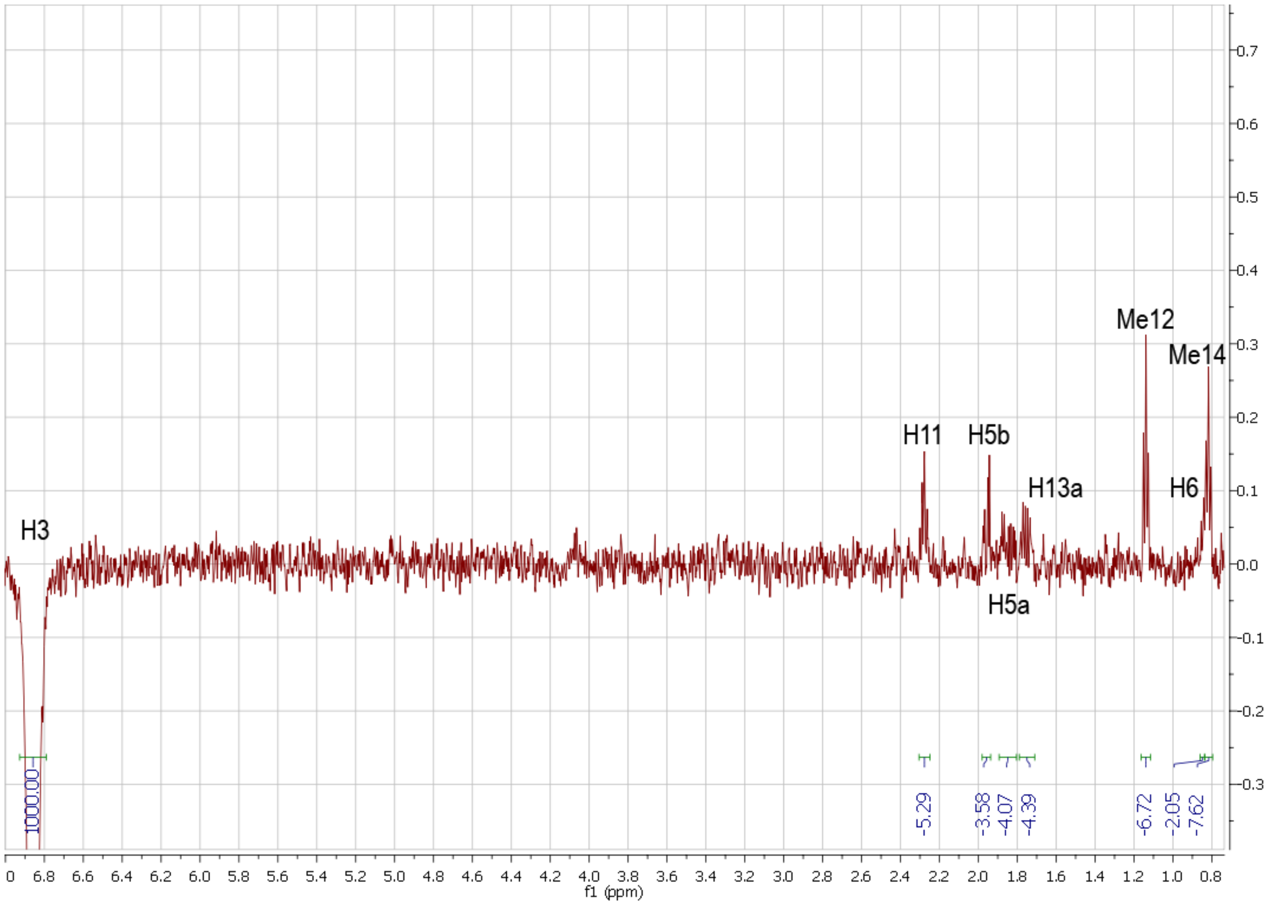

Figure S27: Selective 1D-NOESY spectrum (600 MHz, $CDCl_3$) of H3 of plakilactone H (**2**).

Table S2: Experimental (in $CDCl_3$) and calculated (in vacuo) ^{13}C chemical shifts of diastereoisomers **2a,b,e,f**.

		**2a**	**2b**	**2e**	**2f**
carbon	δ_{exp}	δ_{calc}	δ_{calc}	δ_{calc}	δ_{calc}
10	9.7	10.8	9.6	9.6	10.8
9	24.9	25.7	25.6	26.0	25.1
8	60.9	58.5	61.0	59.1	62.1
7	62.1	59.5	60.6	60.5	61.1
6	38.3	36.1	37.9	38.8	36.4
5	39.2	36.7	40.3	40.6	39.3
4	89.2	86.7	87.5	86.6	87.4
1	173.6	169.3	169.9	169.2	170.5
2	136.1	138.8	139.8	137.3	140.4
3	150.2	151.0	150.7	153.5	150.0
11	18.3	20.3	20.5	20.4	20.6
12	11.8	10.0	10.6	10.4	10.5
13	31.2	30.7	32.2	29.1	32.6
14	7.7	7.9	6.9	7.4	6.8
15	25.8	28.2	27.1	27.1	26.1
16	11.4	10.0	11.4	10.1	8.9

Table S3: Experimental (in $CDCl_3$) and calculated (in vacuo) ^{13}C chemical shifts of diastereoisomers **1a–d**.

		**1a**	**1b**	**1c**	**1d**
carbon	δ_{exp}	δ_{calc}	δ_{calc}	δ_{calc}	δ_{calc}
10	10.1	10.0	10.3	10.6	10.2
9	24.0	21.6	26.2	27.5	22.1
8	73.6	72.4	74.5	73.1	70.7
7	75.6	76.1	71.8	79.3	76.5
6	35.6	36.0	37.2	34.2	34.0
5	36.2	39.2	37.0	37.5	37.0
4	89.6	87.1	87.2	88.8	87.5
1	173.4	169.0	169.5	172.1	169.2
2	135.7	137.8	137.5	140.7	140.4
3	151.2	153.2	153.7	151.4	151.5
11	18.5	20.8	20.9	20.6	20.7
12	12.0	11.1	11.3	11.4	11.0
13	30.9	30.3	28.2	32.2	32.6
14	7.8	7.3	7.3	7.2	7.4
15	25.3	24.2	22.2	20.4	24.5
16	10.7	9.6	11.2	11.8	10.4

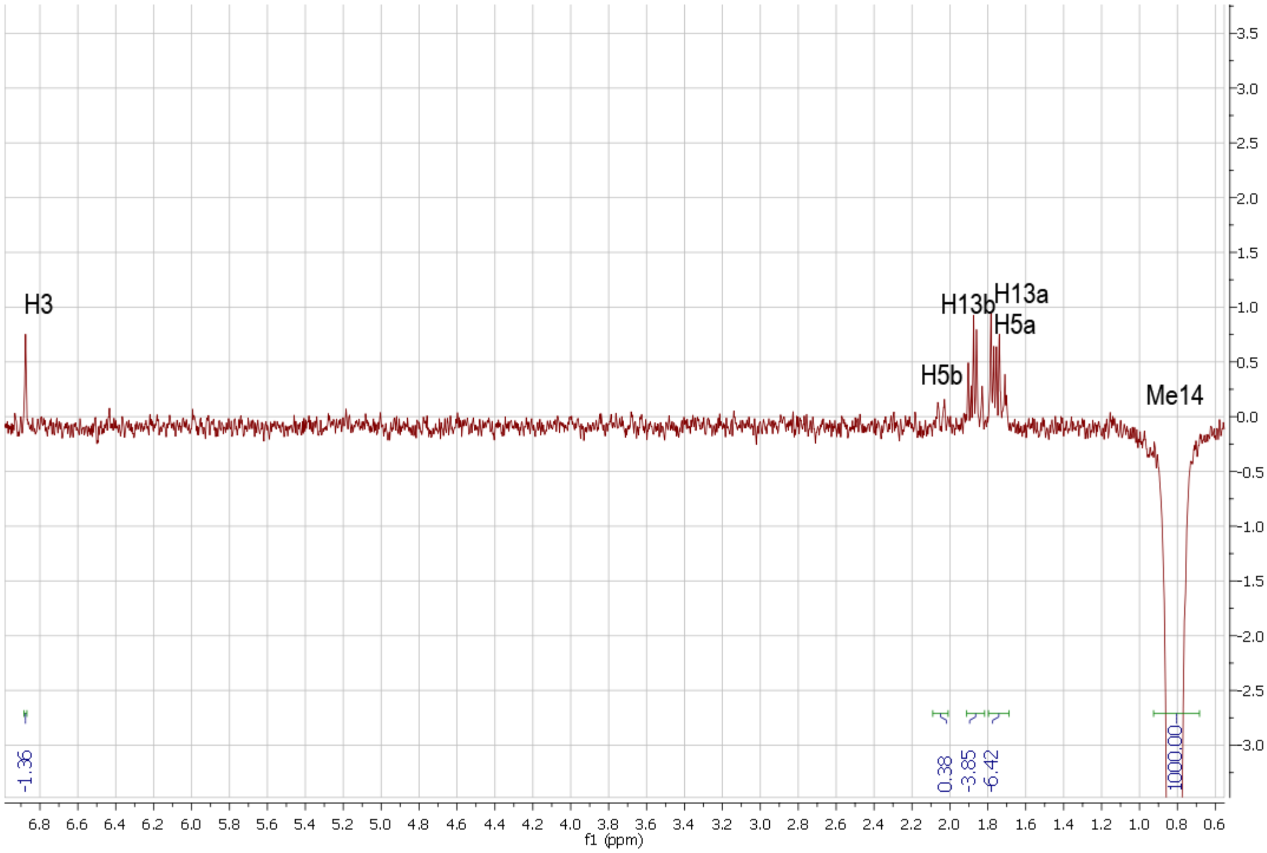

Figure S28: Selective 1D-NOESY spectrum (500 MHz, $CDCl_3$) of Me14 of plakilactone G (**1**).

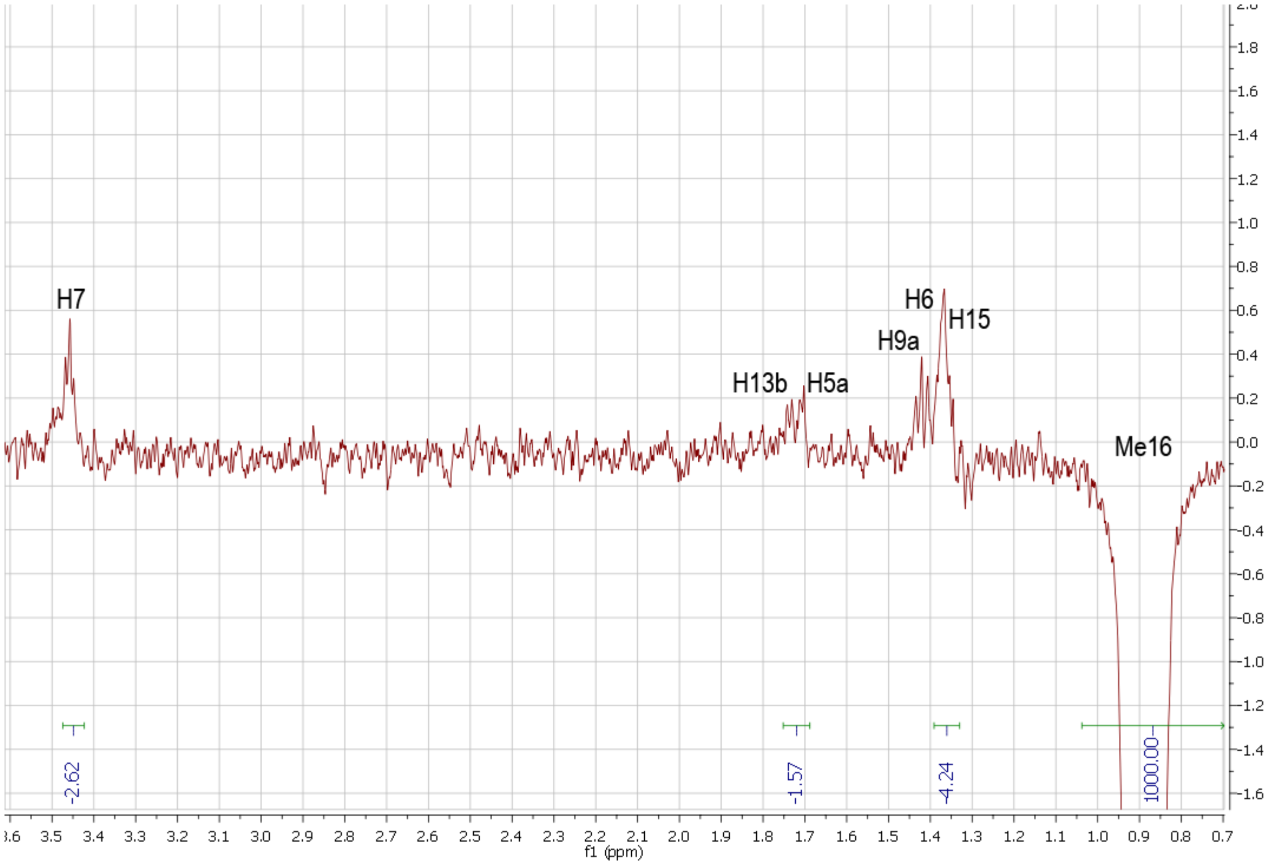

Figure S29: Selective 1D-NOESY spectrum (500 MHz, $CDCl_3$) of Me16 of plakilactone G (**1**).

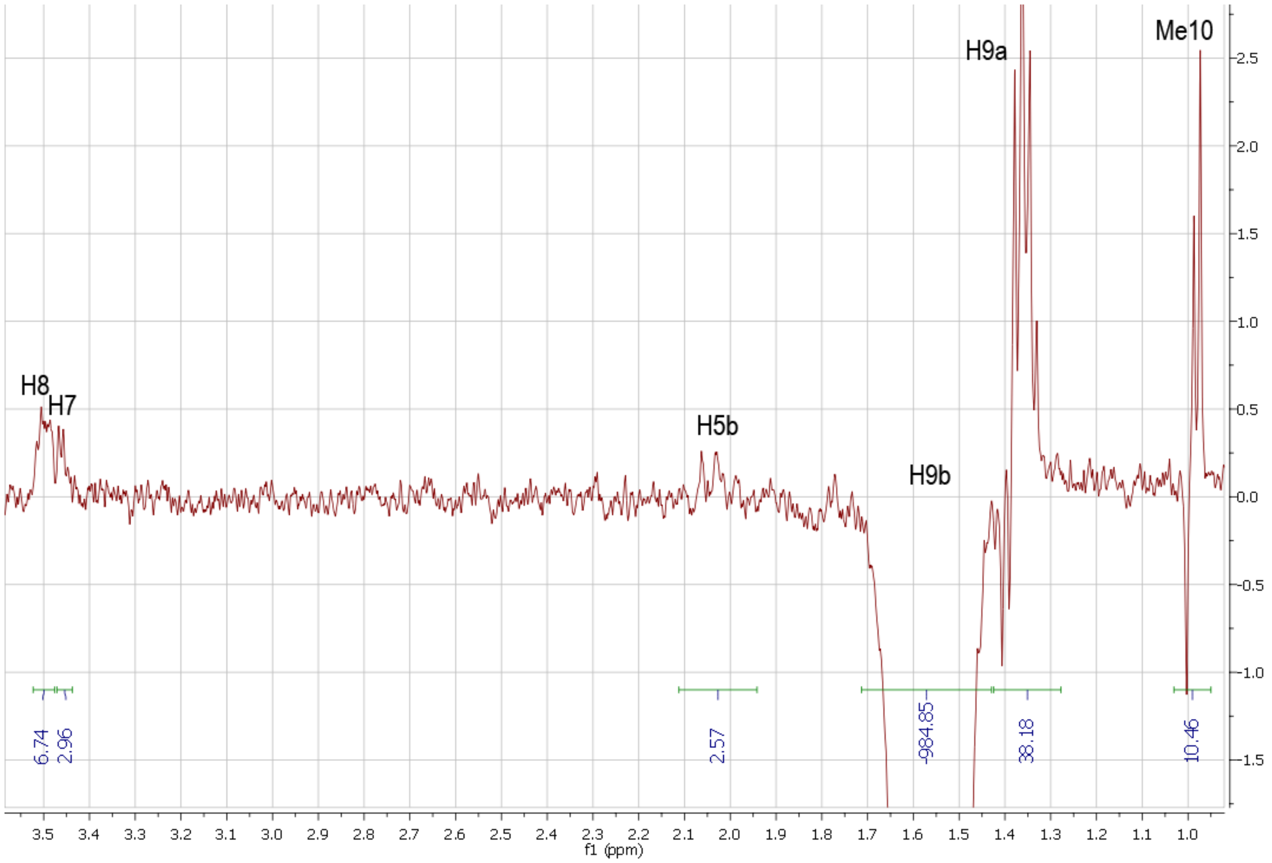

Figure S30: Selective 1D-NOESY spectrum (500 MHz, $CDCl_3$) of H9b of plakilactone G (**1**).

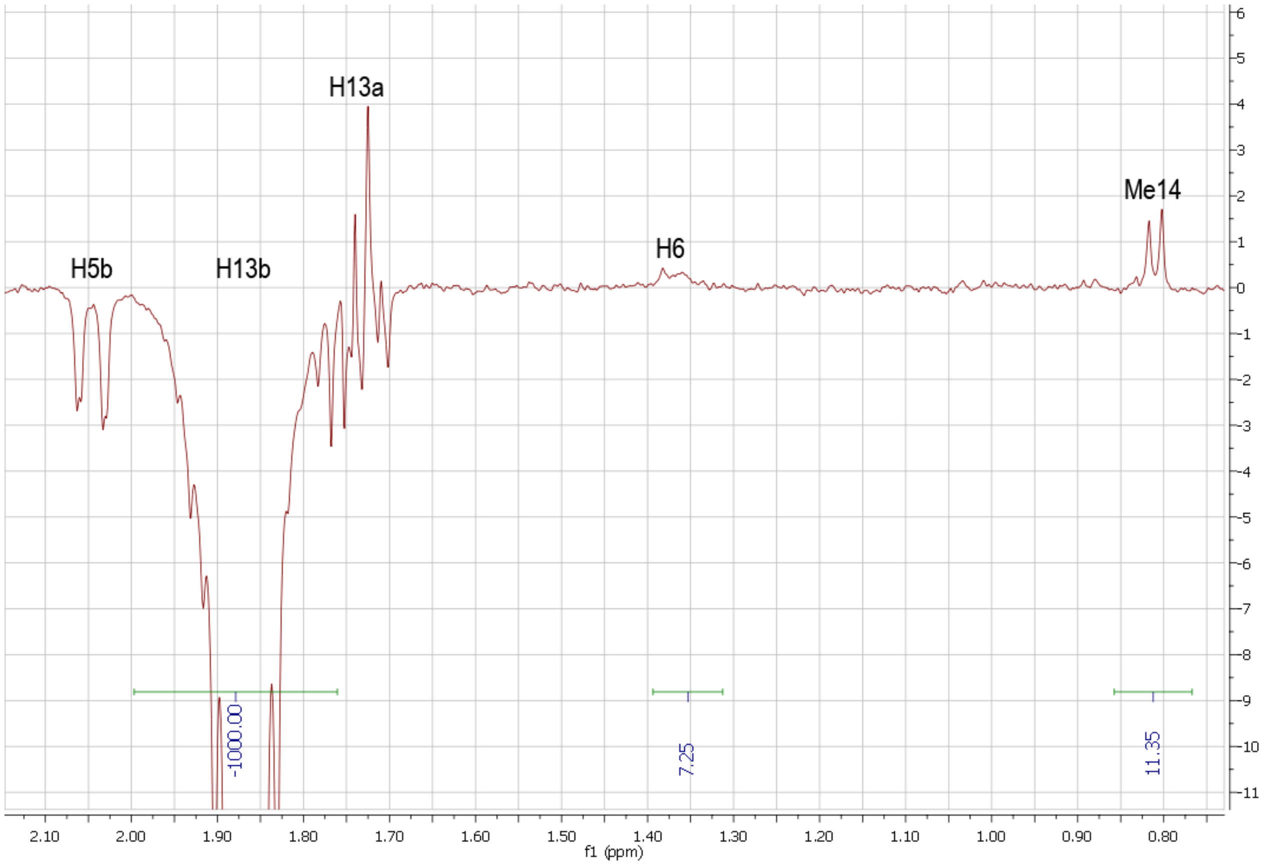

Figure S31: Selective 1D-NOESY spectrum (500 MHz, $CDCl_3$) of H13b of plakilactone G (**1**).

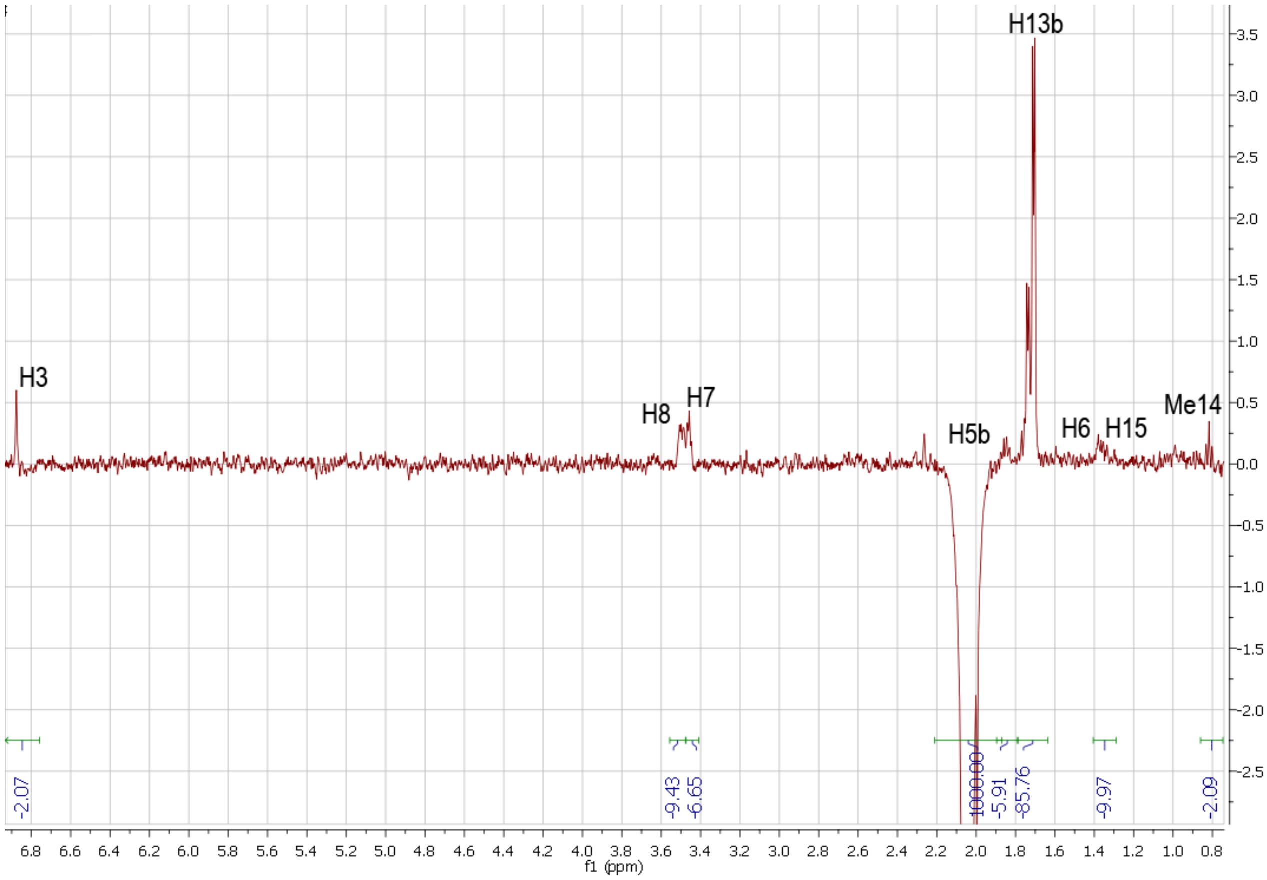

Figure S32: Selective 1D-NOESY spectrum (500 MHz, $CDCl_3$) of H5b of plakilactone G (**1**).

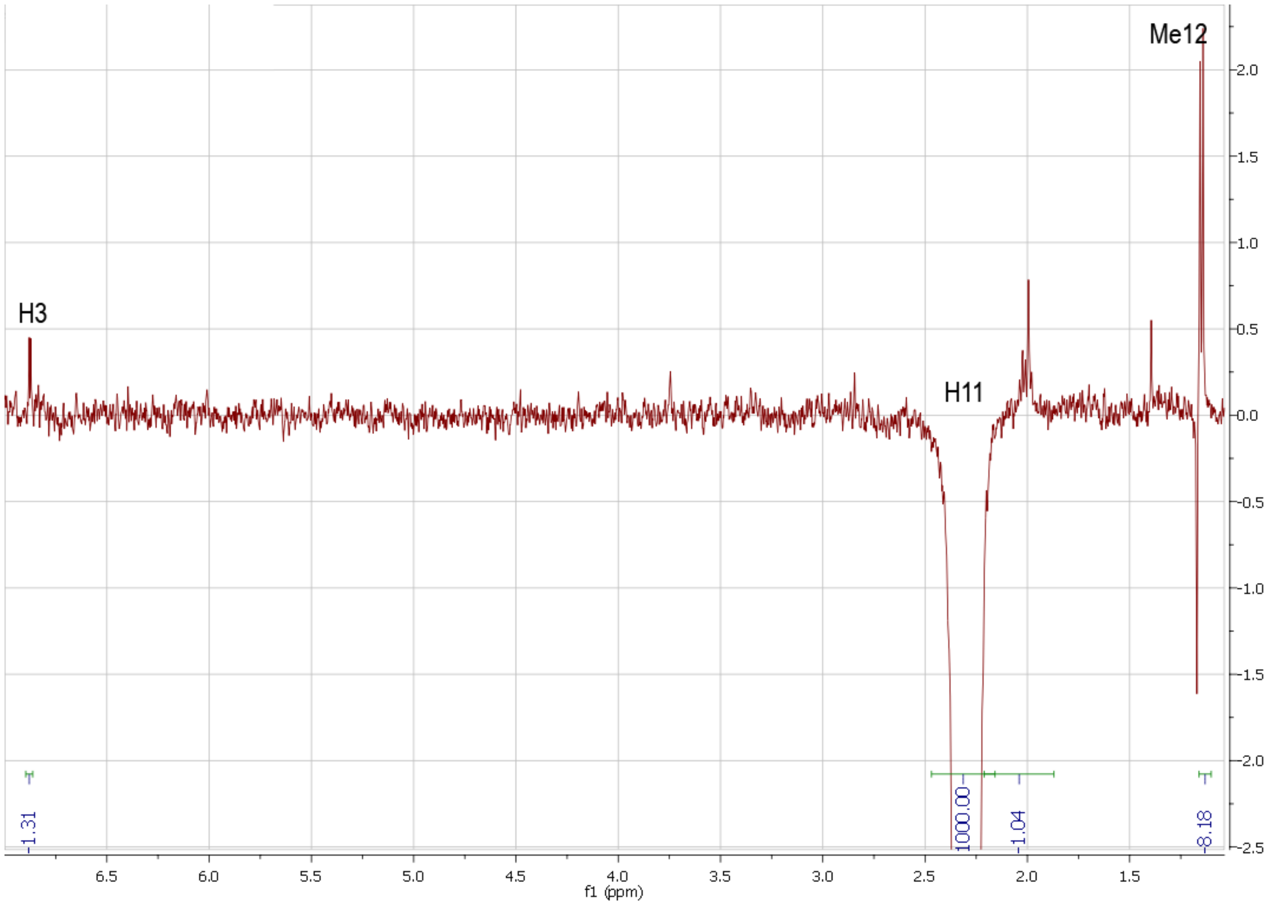

Figure S33: Selective 1D-NOESY spectrum (500 MHz, $CDCl_3$) of H11 of plakilactone G (**1**).

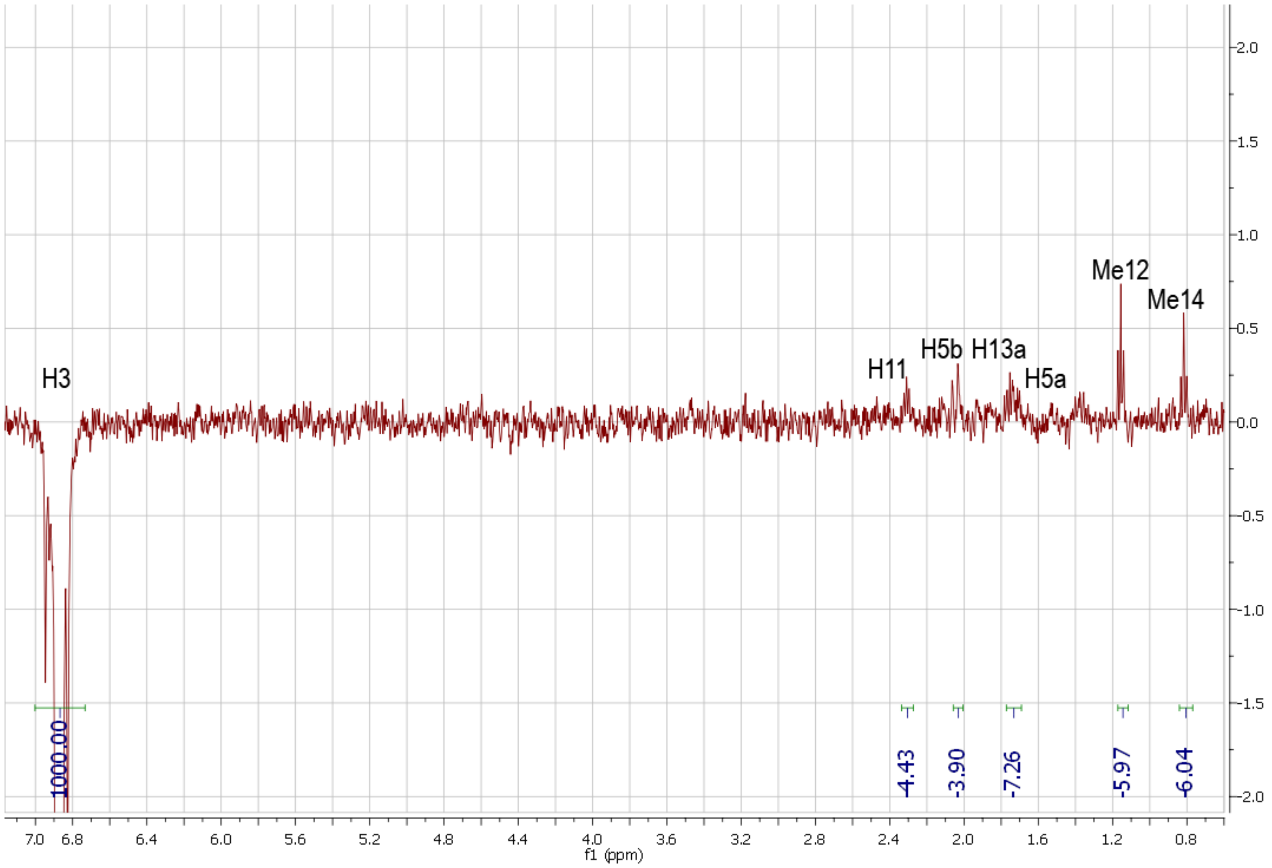

Figure S34: Selective 1D-NOESY spectrum (500 MHz, $CDCl_3$) of H3 of plakilactone G (**1**).

Table S4: Comparison of inter-proton distances determined by NOEs for plakilactone G (**1**) in $CDCl_3$ with DFT-calculated value for **1a–d**. The value in bold was used to calibrate the NOEs.

Proton		1a			1b			1c			1d		
		exp R_{NOE} (Å)	R_{calcd} (Å)	ABS % error[a]	exp R_{NOE} (Å)	R_{calcd} (Å)	ABS % error[a]	exp R_{NOE} (Å)	R_{calcd} (Å)	ABS % error[a]	exp R_{NOE} (Å)	R_{calcd} (Å)	ABS % error[a]
H5b	H5a	**1.69**	**1.76**	4%	**1.73**	**1.76**	0%	**1.64**	**1.76**	7%	**1.68**	**1.76**	5%
H5b	H3	2.99	2.94	1%	3.06	3.13	2%	2.90	2.77	5%	2.97	3.84	26%
H5b	H7	2.59	2.53	2%	2.65	3.01	13%	2.51	2.50	1%	2.57	2.55	1%
H5b	H13b	2.64	2.76	4%	2.70	2.70	0%	2.56	2.79	9%	2.62	2.68	2%
H5b	H15	2.42	2.75	13%	2.48	2.81	13%	2.35	2.85	19%	2.40	2.75	13%
H3	H11	3.11	3.23	4%	3.18	3.19	0%	3.02	3.25	8%	3.09	3.28	6%
H3	Me12	3.16	3.14	1%	3.24	3.22	1%	3.07	3.14	2%	3.15	3.08	2%
H3	Me14	3.16	3.31	5%	3.23	3.22	0%	3.06	3.35	9%	3.14	3.29	5%
H11	Me12	3.00	2.62	13%	3.07	2.62	16%	2.91	2.63	10%	2.98	2.63	13%
H13b	H6	2.55	2.88	12%	2.61	2.74	5%	2.47	4.56	59%	2.53	4.38	54%
H13b	Me14	2.84	2.62	8%	2.91	2.62	10%	2.75	2.63	5%	2.82	2.62	7%
H9b	H8	2.80	3.03	8%	2.87	2.85	1%	2.72	3.04	11%	2.78	3.06	9%
H9b	H9a	1.87	1.76	6%	2.06	1.76	8%	1.81	1.76	3%	2.00	1.76	5%
H9b	Me10	3.15	2.62	18%	3.22	2.63	20%	3.05	2.63	15%	3.13	2.62	18%
Me16	H7	3.02	3.01	0%	3.09	4.25	32%	2.93	4.61	44%	3.00	3.12	4%
**MAE**				*6.8%*			*8.1%*			*13.8%*			*11.3%*
**STD**				*8.8%*			*12.5%*			*19.9%*			*17.0%*

[a]|% error| = $|R_{calcd} - R_{NOE}|/[(R_{calcd} + R_{NOE})/2]$, absolute differences for calculated versus NOE-derived distances/calculated distances.
[b]MAE = Σ[%error]/n.
